# Cytochrome P450‐catalyzed biosynthesis of furanoditerpenoids in the bioenergy crop switchgrass (*Panicum virgatum* L.)

**DOI:** 10.1111/tpj.15492

**Published:** 2021-09-24

**Authors:** Andrew Muchlinski, Meirong Jia, Kira Tiedge, Jason S. Fell, Kyle A. Pelot, Lisl Chew, Danielle Davisson, Yuxuan Chen, Justin Siegel, John T. Lovell, Philipp Zerbe

**Affiliations:** ^1^ Department of Plant Biology University of California – Davis Davis California 95616 USA; ^2^ Genome Center University of California – Davis Davis California 95616 USA; ^3^ Department of Chemistry University of California – Davis Davis California 95616 USA; ^4^ Department of Biochemistry & Molecular Medicine University of California – Davis Davis California 95616 USA; ^5^ Genome Sequencing Center Hudson Alpha Institute for Biotechnology Huntsville Alabama 35806 USA; ^6^ Present address: Firmenich Inc. 4767 Nexus Center Dr. San Diego California 9212 USA; ^7^ Present address: State Key Laboratory of Bioactive Substance and Function of Natural Medicines & NHC Key Laboratory of Biosynthesis of Natural Products Institute of Materia Medica Chinese Academy of Medical Sciences & Peking Union Medical College Beijing 100050 China

**Keywords:** cytochrome P450 monooxygenase, diterpenoid biosynthesis, plant specialized metabolism, plant natural products, *Panicum virgatum*

## Abstract

Specialized diterpenoid metabolites are important mediators of plant–environment interactions in monocot crops. To understand metabolite functions in plant environmental adaptation that ultimately can enable crop improvement strategies, a deeper knowledge of the underlying species‐specific biosynthetic pathways is required. Here, we report the genomics‐enabled discovery of five cytochrome P450 monooxygenases (CYP71Z25–CYP71Z29) that form previously unknown furanoditerpenoids in the monocot bioenergy crop *Panicum virgatum* (switchgrass). Combinatorial pathway reconstruction showed that CYP71Z25–CYP71Z29 catalyze furan ring addition directly to primary diterpene alcohol intermediates derived from distinct class II diterpene synthase products. Transcriptional co‐expression patterns and the presence of select diterpenoids in switchgrass roots support the occurrence of P450‐derived furanoditerpenoids *in planta*. Integrating molecular dynamics, structural analysis and targeted mutagenesis identified active site determinants that contribute to the distinct catalytic specificities underlying the broad substrate promiscuity of CYP71Z25–CYP71Z29 for native and non‐native diterpenoids.

## Significance Statement

Cytochrome P450 monooxygenases catalyze the various functional decorations of core diterpene scaffolds that determine the large diversity of biologically active plant diterpenoids. This study describes the identification, structure–function analysis and gene expression analysis of a group of cytochrome P450 monooxygenases, CYP71Z25–CYP71Z29, from the bioenergy crop switchgrass (*Panicum virgatum*). CYP71Z25–CYP71Z29 catalyze the addition of a furan ring to primary alcohol derivatives of class II diterpene synthase products.

## INTRODUCTION

Diverse networks of specialized metabolites impact plant fitness by mediating ecological interactions among plants, microbes and animals. Among these metabolites, diterpenoids play essential roles in plant defense and ecological adaptation (Tholl, 2015). For instance, chemically distinct diterpenoid blends confer pathogen and pest resistance in major global grain crops, including *Zea mays* (maize) and *Oryza sativa* (rice) (Ding et al., 2021; Murphy and Zerbe, 2020). Recent studies further suggest diterpenoid functions in abiotic stress adaptation. For example, UV irradiation elicited diterpenoid accumulation and the expression of the corresponding metabolic genes in rice, and rice diterpenoids were shown to impact stomatal closure and drought tolerance in selected cultivars (Horie et al., 2015; Park et al., 2013; Zhang et al., 2021). Inducible diterpenoid formation was also observed in maize in response to oxidative, drought and salinity stress (Christensen et al., 2018; Mafu et al., 2018; Vaughan et al., 2014), and diterpenoid‐deficient maize mutants show decreased resilience to abiotic perturbations (Vaughan et al., 2015). Expanding our knowledge of diterpenoid diversity and associated metabolic genes and pathways across a broader range of monocot crop species can inform molecular breeding and engineering strategies to improve crop environmental adaptation (Bailey‐Serres et al., 2019; Bevan et al., 2017; Ding et al., 2021; Nelson et al., 2018).

**Figure 1:**
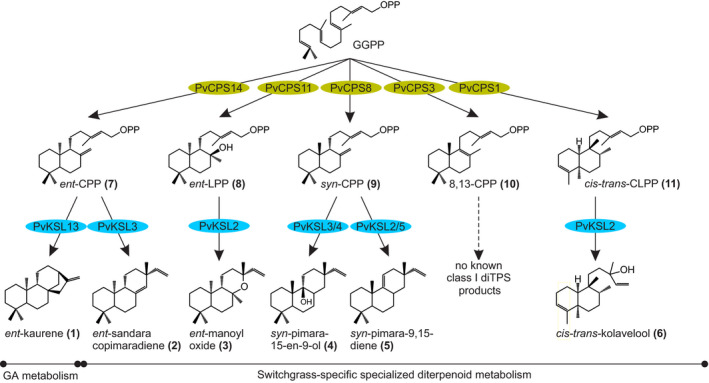
Known switchgrass diterpenoid metabolic network. Shown is an overview of biosynthetic pathways identified in switchgrass. The central diterpenoid precursor, geranylgeranyl pyrophosphate (GGPP) is converted by class II diterpene synthases (diTPSs, light green) to form prenyl pyrophosphate intermediates of distinct stereochemistry and oxygenation. Class I diTPS (blue) typically convert these prenyl pyrophosphate intermediates through cleavage of the pyrophosphate group and downstream rearrangements to yield a range of diterpene scaffolds. These scaffolds can then be further functionally modified by cytochrome P450 monooxygenases (P450s) to form various diterpenoids in general (gibberellin phytohormone) and specialized diterpenoid metabolism.

*Panicum virgatum* (switchgrass) is a key species of the North American tallgrass prairie ecosystem and is valued as a forage and biofuel crop for its high net energy yield and abiotic stress tolerance (Liu et al., 2015; Lovell et al., 2021; Schmer et al., 2008). Broad drought‐induced alterations in carbohydrate, lipid, phenylpropanoid and terpenoid metabolism support a role of specialized metabolites in switchgrass abiotic stress resilience (Li et al., 2021; Meyer et al., 2014; Muchlinski et al., 2019; Pelot et al., 2018). Diterpenoids in monocot crops almost invariably belong to the group of labdane‐related metabolites, and feature species‐specific structures, bioactivities, and spatiotemporal regulation and distribution (Murphy and Zerbe, 2020; Schmelz et al., 2014; Zi et al., 2014). Diterpene synthases (diTPSs) and cytochrome P450 monooxygenases (P450) are the key gatekeepers to diterpenoid diversity (Banerjee and Hamberger, 2018; Karunanithi and Zerbe, 2019). Rooted in the common C20 precursor, geranylgeranyl pyrophosphate (GGPP), the conserved pathway architecture en route to labdane‐related diterpenoids, recruits the combined activity of class II and class I diTPSs. After class II diTPSs catalyzed the conversion of GGPP into bicyclic prenyl pyrophosphate compounds of distinct stereochemistry and oxygenation, class I diTPSs facilitate the dephosphorylation and subsequent cyclization and/or rearrangement of these intermediates to generate various diterpenoid scaffolds (Peters, 2010). Functional decoration through the activity of P450s and other modifying enzyme classes then expands the structural complexity and bioactivity of plant diterpenoids (Banerjee and Hamberger, 2018). Over the past decades, numerous diterpenoid‐metabolic diTPSs and P450s have been identified in maize, rice and *Triticum aestivum* (wheat) (reviewed in Ding et al., 2021; Murphy and Zerbe, 2020; Schmelz et al., 2014), and demonstrated that, downstream of the central GGPP precursor, labdane diterpenoid biosynthesis is organized as modular metabolic grids, where pairwise reactions of functionally distinct enzymes create multiple pathway branches to readily increase product diversity (Ding et al., 2019; Mafu et al., 2018; Morrone et al., 2011; Murphy et al., 2018; Xu et al., 2007). By integrating genome‐wide pathway discovery and combinatorial protein biochemical tools, our prior work identified a large and diverse diTPS family in switchgrass (Pelot et al., 2018) that yields an expansive diversity of diterpenoids. This includes several labdane‐related compounds that, to current knowledge, occur uniquely in switchgrass (Pelot et al., 2018) (Figure 1). The endogenous accumulation of several metabolites and the expression of the corresponding biosynthetic genes in roots and leaves following abiotic stress support a role of terpenoids in switchgrass environmental adaptation (Muchlinski et al., 2019; Pelot et al., 2018); however, complete metabolic pathways, products and their physiological functions remain to be resolved.

Combining genomic studies, combinatorial enzyme assays, metabolite and transcript profiling, and protein structure–function studies revealed a group of five P450s of the CYP71 clan (CYP71Z25–CYP71Z29) that convert a range of diterpene scaffolds into furanoditerpenoid derivatives. The P450‐catalyzed addition of a furan ring directly to diterpene alcohol intermediates derived from class II diTPS activity suggests a possible alternative to the common labdane diterpenoid formation, requiring the pairwise activity of class II and class I diTPSs. Co‐expression of functionally compatible *diTPS*s and *P450*s and presence of key diTPS and P450 products in drought‐stressed switchgrass roots support the presence of these pathway branches *in planta*. Mechanistic insight into CYP71Z25–CYP71Z29 catalysis enables resources for engineering a broad range of bioactive furanoditerpenoids.

## RESULTS

### Identification of diterpenoid metabolic P450s in the switchgrass genome

**Figure 2:**
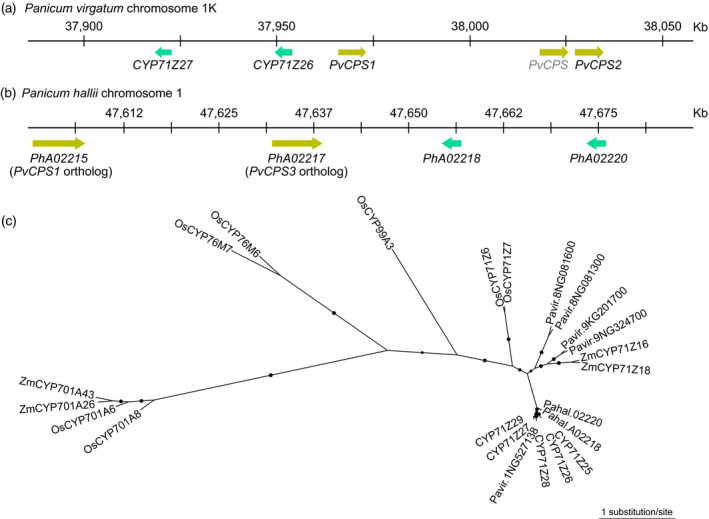
Discovery of switchgrass (*Panicum virgatum*) cytochrome P450 monooxygenases. (a, b) Genomic co‐location of P450 genes (red) with class II diterpene synthases (blue) in the *P. virgatum* (var. Alamo AP13) genome (v5.1) and the genome of the diploid switchgrass relative *Panicum hallii* (var. filipes FIL2, v3.1) (Lovell et al., 2018). (c) Unrooted maximum‐likelihood phylogenetic tree of CYP71Z25–CYP71Z29 (for the corresponding gene IDs, see Table S4) with predicted or characterized diterpenoid metabolic P450s from other monocot crops (Table S4). Branches with bootstrap support greater than 80% (1000 repetitions) are depicted by black circles.

To elucidate P450 pathways for the functional decoration of the expansive spectrum of diterpenoid structures in switchgrass, we probed the genomic regions neighboring previously characterized switchgrass *diTPS* genes in the *P. virgatum* var. Alamo AP13 genome (v5.1) (Lovell et al., 2021; Pelot et al., 2018). A tandem pair of two *P450* genes, *Pavir.1KG382300* (*CYP71Z26*) and *Pavir.1KG382400* (*CYP71Z27*), co‐localized on chromosome 1K in direct proximity to three class II *diTPS* genes, including the *cis‐trans*‐clerodienyl pyrophosphate (*cis‐trans*‐CLPP) synthase *PvCPS1* (*Pavir.1KG382200*) (12.5 kb and 43.5 kb, respectively), its paralog *PvCPS2* (*Pavir.1KG382115*) and an additional predicted *diTPS* (*Pavir.1KG382110*) (Figure 2a). An additional P450 candidate, *Pavir.1KG341400* (*CYP71Z25*), was identified distantly (1.3 Mb from *CYP71Z27*) on chromosome 1K. Further homology‐based mining of the switchgrass genome with the above genes as bait identified seven additional gene candidates with significant matches to P450s of the CYP71Z subfamily. *Pavir.1NG304500* (*CYP71Z28*), *Pavir.1NG309700* (*CYP71Z29*) and *Pavir.1NG527138* are located distantly (>500 kb) from each other on chromosome 1N. Of the remaining gene candidates, *Pavir.8NG081300*, *Pavir.8NG081600*, *Pavir.9KG201700* and *Pavir.9NG324700*, only *Pavir.9KG201700* featured all conserved P450 sequence recognition site (SRS) motifs likely encoding a functional P450. Orthology network analysis including the genomes of switchgrass, the diploid switchgrass relative *Panicum hallii* (Lovell et al., 2018) and *Setaria italica* (foxtail millet) (Bennetzen et al., 2012) identified one CYP71Z25–CYP71Z29 paralog in foxtail millet and two possible paralogs in *P. hallii* (Figure S1). Notably, the paralogs *Pahal.A02218* and *Pahal.A02220* were also clustered in the genome of *P. hallii* (var. filipes FIL2; v2.0) (Lovell et al., 2018) and were co‐localized with two class II diTPSs, *Pahal.A02215* and *Pahal.A02217*, with predicted *cis‐trans*‐CLPP and 8,13‐CPP synthase activities, respectively (Pelot et al., 2018) (Figure 2b). Phylogenetic analysis with characterized diterpenoid metabolic P450s from related Poaceae species placed *Pavir.9KG201700* most closely to maize CYP71Z16/18, with functions in specialized diterpenoid biosynthesis (Ding et al., 2019; Kitaoka et al., 2015a; Mafu et al., 2018; Mao et al., 2016; Wu et al., 2011) (Figure 2c). By contrast, CYP71Z25–CYP71Z29 clustered in a separate clade together with the *P. hallii* paralogs, indicating likely species‐specific functionalities, and hence were chosen for biochemical analysis in this study. *Pavir.1NG527138* showed >99% amino acid sequence identity to CYP71Z28, with no differences among the six SRS motifs, suggesting catalytic redundancy. Therefore, *CYP71Z25* (*Pavir.1KG341400*), *CYP71Z26* (*Pavir.1KG382300*), *CYP71Z27* (*Pavir.1KG382400*), *CYP71Z28* (*Pavir.1NG304500*) and *CYP71Z29* (*Pavir.1NG309700*) were selected for further functional analysis.

### Switchgrass CYP71Z enzymes form distinct furanoditerpenoids

**Figure 3:**
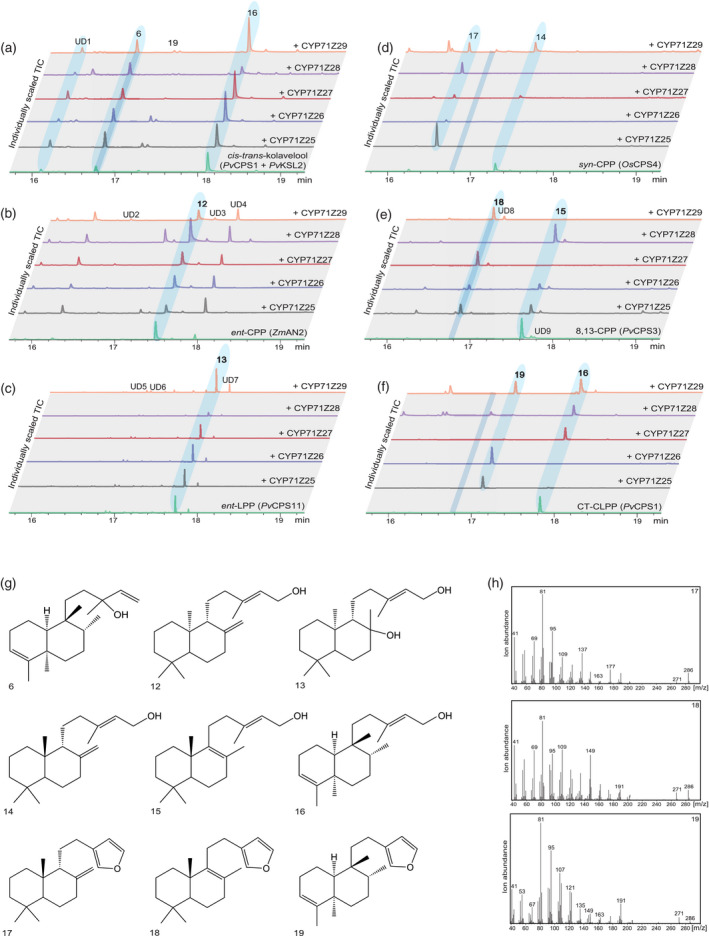
Functional characterization of switchgrass CYP71Z25–CYP71Z29. (a–f) Individually scaled total ion GC‐MS chromatograms of enzyme products resulting from *Escherichia coli* co‐expression of each P450 candidate (CYP71Z25–CYP71Z29) with different diterpene synthases: (a) *Pv*CPS1 and *Pv*KSL2 producing the diterpenoid scaffold *cis*‐*trans*‐kolavelool (**6**); (b) ZmAN2 producing *ent*‐copalol (**12**) (dephosphorylated *ent*‐copalyl pyrophosphate (CPP), **7**); (c) PvCPS11 producing *ent*‐labda‐13*E*‐en‐8α,15‐ol (**13**) (dephosphorylated *ent*‐labdadienyl pyrophosphate (LPP), **8**); (d) *Oryza sativa* CPS4 producing *syn*‐copalol (**14**) (dephosphorylated *syn‐*CPP, **9**); (e) PvCPS3 producing 8,13‐copalol (**15**) (dephosphorylated 8,13‐CPP, **10**); and (f) PvCPS1 producing *cis‐trans*‐kolavenol (**16**) (dephoshorylated *cis*‐trans‐clerodienyl pyrophosphate (CT‐CLPP), **11**). Compounds labeled as UD1–UD9 indicate unidentified terpenoid products based on mass spectral patterns. Unlabeled peaks represent non‐terpenoid compounds derived from *E. coli* expression cultures. (g) Structures of relevant enzyme products. Structures of **17**, **18** and **19** were verified by NMR analysis and absolute stereochemistry assigned based on the corresponding class II diterpene synthase products. (h) Mass spectra of identified P450 furanoditerpenoid products.

To functionally characterize CYP71Z25–CYP71Z29, we employed combinatorial pathway reconstruction assays of codon‐optimized, N‐terminally modified P450 constructs with functionally distinct diTPSs and a maize cytochrome P450 reductase (*Zm*CPR2) using an established *Escherichia coli* expression platform (Cyr et al., 2007; Morrone et al., 2010). Following the typical pathway organization of plant labdane diterpenoid metabolism (Peters, 2010; Zi et al., 2014), we first tested the co‐expression of each P450 with different pairs of class II and class I diTPSs that produce six core diterpenoid scaffolds formed by the switchgrass diTPS family (Pelot et al., 2018), namely *ent*‐kaurene (**1**) (derived from *ent*‐copalyl pyrophosphate, CPP, **7**), *ent*‐sandaracopimaradiene (**2**) (derived from **7**), *ent*‐manoyl oxide (**3**) (derived from *ent*‐labdadienyl pyrophosphate, LPP, **8**), *syn*‐pimara‐15‐en‐9‐ol (**4**) (derived from *syn*‐CPP, **9**), *syn*‐pimara‐9,15‐diene (**5**) (derived from **9**) and *cis‐trans*‐kolavelool (**6**) (derived from *cis‐trans*‐clerodienyl pyrophosphate, CLPP, **11**). For compound numbering and structures see Figure 1 and Figure S2. When compared with the products of the combined class II and class I diTPS activity alone, trace quantities of P450 products were detected only for CYP71Z25, CYP71Z26 and CYP71Z29 when co‐expressed with *Pv*CPS1 and *Pv*KSL2 that form **11** and **6**, respectively (Figure 3a; Figure S3). These P450 products featured near identical fragmentation patterns with characteristic mass ions of *m*/*z* 286, 191, 177, 95 and 81, indicative of an oxygenated labdane scaffold. Notably, the presence of these P450 products when co‐expressed with *Pv*CPS1 and *Pv*KSL2 coincided with a substantially lower diTPS product yield, as compared with all other diTPS combinations tested, probably through the lower catalytic efficiency of *Pv*KSL2. As a result of this *Pv*KSL2 inefficiency, a higher abundance of *cis‐trans‐*kolavenol (**16**) was observed, presumably resulting from the conversion of the *Pv*CPS1 product *cis‐trans*‐CLPP (**11**) by endogenous *E. coli* phosphatases. Given the presence of P450 products in a scenario of class I diTPS inefficiency, it was conceivable that CYP71Z25–CYP71Z29 act more directly on the class II diTPS products or their respective dephosphorylated derivatives. This hypothesis is supported by the genomic co‐localization of several P450 candidates with *PvCPS1* or *PvCPS2* (Figure 2). To test this hypothesis, *E. coli* co‐expression assays were conducted pairing each P450 candidate with individual class II diTPSs that produce the distinct prenyl pyrophosphate products known in switchgrass: **7**, **8**, **9**, **11** and 8,13‐CPP (**10**) (Pelot et al., 2018) (Figure S2). Gas chromatography–mass spectrometry (GC‐MS) analysis of the respective reaction products showed product formation by all P450s, although with apparent differences in substrate specificity (Figure 3b–f). It should be noted that GC‐MS analysis detected the dephosphorylated class II diTPS products through the activity of endogenous *E. coli* phosphatases (*ent*‐copalol, **12**; *ent*‐labda‐13*E*‐en‐8α,15‐ol, **13**; *syn*‐copalol, **14**; 8,13‐copalol, **15**; and *cis‐trans*‐kolavenol, **16**; Figure S2). No P450 products were observed with **12** or **13** as substrates (Figure 3b,c). The conversion of **16** was observed for CYP71Z25, CYP71Z26 and CYP71Z29, whereas CYP71Z27 and CYP71Z28 showed no activity. Importantly, the resulting P450 product featured the same retention time and a near‐identical fragmentation pattern as compared with the product observed when combing the *cis‐trans*‐CLPP (**11**) synthase *Pv*CPS1 with the *cis‐trans*‐kolavelool (**6**) synthase *Pv*KSL2 and a P450 candidate (Figure 3a,f). The conversion of **15** was observed for CYP71Z27 and CYP71Z29, and partially for CYP71Z25 and CYP71Z26, whereas CYP71Z28 was largely inactive with this substrate (Figure 3e). By contrast, CYP71Z28 showed high activity with **14** as a substrate, as did CYP71Z25 and CYP71Z26, whereas CYP71Z27 and CYP71Z29 showed only incomplete substrate conversion (Figure 3d). For all observed P450 products, fragmentation patterns featured *m*/*z* 286, 191 and 177 mass ions, consistent with oxygenated labdane diterpenoid structures (Figure 3g,h).

To determine the structure of selected P450 products, enzymatically produced and purified compounds were subject to 1D and 2D NMR (HSQC, COSY, HMBC and NOESY) analysis and compared with previously reported NMR data, where available. This approach revealed a shared structural scaffold of the P450 products that contains the individual class II diTPS‐derived diterpene backbones with the addition of a furan ring at the C15–C16 position (Figure 3g). Furanoditerpenoid products identified here included *syn*‐15,16‐epoxy‐8(17),13(16),14‐triene (**17**) derived from the *Pv*CPS8 product (**14**) (Figure S4), 15,16‐epoxy‐8,13(16),14‐triene (**18**) derived from the *Pv*CPS3 product (**15**) (Figure S5) and *cis‐trans*‐15,16‐epoxy‐cleroda‐3,13(16),14‐triene (**19**) derived from the *Pv*CPS1 product (**16**) (Figure S6). The stereochemistry of the P450 products was assigned on the basis of the relevant class II diTPS products (Pelot et al., 2018).

Considering the substrate promiscuity of CYP71Z25–CYP71Z29, we next investigated the capacity of these P450s to convert alternative class II diTPS products. Indeed, with the exception of CYP71Z28, all P450s formed (+)‐15,16epoxy‐8(17),13(16),14‐triene (**26**) when co‐expressed with a diTPS producing (+)‐CPP (**20**) and its alcohol derivative (+)‐copalol (**23**), class II diTPS products not currently known in switchgrass, but formed by diTPSs of other monocot crops such as wheat and maize (Murphy et al., 2018; Wu et al., 2012) (Figures S7 and S8). In addition, CYP71Z25 and CYP71Z26 showed, albeit low, activity when co‐expressed with the *trans‐cis*‐neo‐clerodienyl pyrophosphate (*trans‐cis*‐CLPP, **22**; *trans‐cis*‐kolavenol, **25**) synthase of *Salvia divinorum*, *Sd*CPS2 (Pelot et al., 2017), forming the core precursor to salvinorin A, a neoclerodane furanoditerpenoid with potential use for the treatment of drug addiction and neuropsychiatric disorders (Kivell et al., 2014). The two major P450 products showed signature mass ions of *m*/*z* 286 or *m*/*z* 288, indicating a **25**‐derived furanoditerpenoid (**28**) and *trans*‐*cis*‐cleroda‐3,12‐dien‐15,16‐diol (**29**) structures, respectively (Figure S7).

The formation of furanoditerpenoids through the coupled activity of CYP71Z25–CYP71Z29 and functionally distinct class II diTPSs warranted a deeper investigation into the nature of the P450 substrate. Whereas class II diTPSs form prenyl pyrophosphate products with a characteristic pyrophosphate group at C15 (Peters, 2010), as mentioned above the expression of class II diTPSs in heterologous plant or microbial host systems typically yields the corresponding primary alcohol derivatives formed through the activity of endogenous phosphatases (Ding et al., 2019; Mafu et al., 2018; Pelot et al., 2018). To examine the catalytic preferences of CYP71Z25–CYP71Z29, substrate feeding experiments were conducted by adding enzymatically produced and purified compounds of **15** or **16** to *E. coli* cultures expressing CYP71Z25 or CYP71Z29. For both P450–substrate combinations tested, conversion of the 15‐hydroxy diterpene substrates into the respective furanoditerpenoids was observed, whereas no conversion was detected in control samples expressing plasmids carrying *Zm*CPR2 alone (Figure S9). Substantial limitations in purifying the corresponding prenyl pyrophosphate compounds prevented us from conducting complementary feeding assays within the scope of this study.

### Structure‐guided mutagenesis identifies active site determinants of P450 catalytic specificity

To investigate the catalytic mechanism underlying the activity of CYP71Z25–CYP71Z29, homology models were generated for each P450 using the recently reported crystal structure of *Salvia miltiorrhiza* CYP76AH1 (Gu et al., 2019) as a template. Given a protein identity of only approximately 42% to the target P450s (Figure S10), iterative homology modeling with an energy minimization approach employing relaxed template protein structures (Nivón et al., 2013; Pei and Grishin, 2014) was used to generate high‐quality models. The resulting lowest energy model was used for ligand docking of the heme co‐factor into the individual active sites. The structural models generated showed root‐mean‐square deviation (RMSD) values of 0.67–0.75, as compared with the template, thus representing the high‐quality reproduction of common secondary structures and placement of active site residues, as demonstrated, for example, for the heme‐anchoring cysteine C421 (Figure S10). The five diterpenoid substrates tested in this study were then docked into the heme model complex with three conceivable substitution arrangements: 15‐hydroxy and 15‐pyrophosphate structures as alternative substrates and 15,16‐dihydroxy derivatives as intermediate or product of the P450‐catalyzed oxygenation reaction (Figures S2 and S10). A comparison of the interaction energy (IE) of all substrate–P450 docking poses generated showed that the average IE was most favorable for the 15‐hydroxy and 15‐16‐dihydroxy substrates, whereas the 15‐pyrophosphate structure was energetically less favorable (Table S1). The difference in IE between the 15‐hydroxy and 15‐pyrophosphate ligands can presumably be attributed to the far larger volume and electrostatic charge of the pyrophosphate moiety.

**Figure 4:**
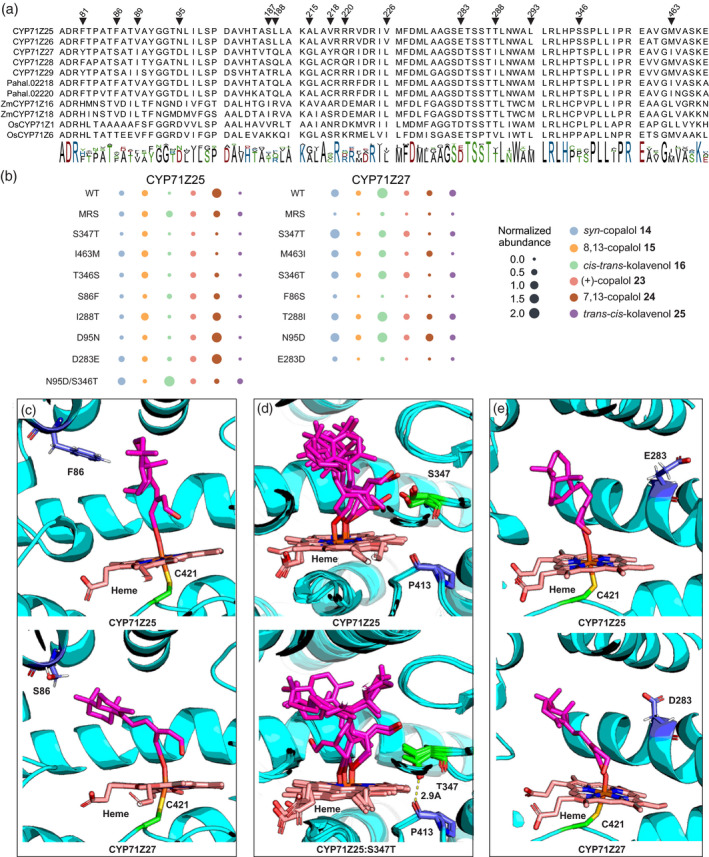
(a) Protein sequence alignment of substrate recognition site (SRS) motifs of CYP71Z25–CYP71Z29 and related members of the CYP71Z subfamily of known or predicted function. (b) Analysis of standardized substrate conversion by CYP71Z25 and CYP71Z27 protein variants. *Escherichia coli* co‐expressions for each variant with native (**14**, **15**, **16**) and non‐native (**23**, **24**, **25**) switchgrass substrates were carried out in triplicate and normalized to the internal standard 1‐eicosane and the OD_600_ at the time of the induction of protein expression. Error bars indicate the standard deviations of replicates from the mean. MRS indicates the reciprocal mutagenesis of all 15 identified residues between CYP71Z25 and CYP71Z27 (F/Y81, F/S86, V/I89, N/D95, S/T187, L/Q188, A/G215, V/Y218, R/Q220, V/L226, E/D283, T/I288, L/M293, S/T346, M/I463). (c) Active sites of CYP71Z25 (top) and CYP71Z27 (bottom) with heme (magenta), substrate (pink), and residues C412 (green) and S/F86 (blue). (d) Structural overlay of the five lowest interaction energy (IE) active sites of CYP71Z25 (top) and CYP71Z25‐S347T variant (bottom) with **15** (8,13‐copalol) substrate (pink) and heme (magenta) docked. Residues S/T347 (green), P413 (blue, and the corresponding hydrogen bonding (yellow dashes), with distance in Ångstrom, are highlighted). (e) Active sites of CYP71Z25 (top) and CYP71Z27 (bottom) with heme (magenta), substrate (pink), and residues C412 (green) and E/D283 (blue).

The various modeled substrate–protein complexes were then used to investigate active site residues with possible impact on the distinct substrate specificity of CYP71Z25–CYP71Z29. A total of 15 active site residues associated with the six known CYP71 SRSs (Dueholm et al., 2015) were identified and were located proximal to the docked substrates, and showed residue variation among CYP71Z25–CYP71Z29 and related members of the CYP71Z subfamily (Figure 4a). To investigate the catalytic impact of these residues, protein variants of CYP71Z25 and CYP71Z27, as the functionally most contrasting P450s, were generated via site‐directed mutagenesis and functionally characterized by *E. coli* co‐expression with individual class II diTPSs producing native (**14**, **15**, **16**) or non‐native (**23**, **24**, **25**) P450 substrates. Reciprocal mutagenesis of all 15 residues identified between CYP71Z25 and CYP71Z27 (F/Y81, F/S86, V/I89, N/D95, S/T187, L/Q188, A/G215, V/Y218, R/Q220, V/L226, E/D283, T/I288, L/M293, S/T346 and M/I463) resulted in a near‐complete loss of function of the corresponding CYP71Z25 variant (Figure 4b, Figure S10). By contrast, the reciprocal multi‐residue variant of CYP71Z27 featured an altered product profile largely reflecting the wild‐type products of CYP71Z25. Strikingly, the single‐residue variant CYP71Z27:S86F showed a product profile similar to that observed for the multi‐residue variant of CYP71Z27, converting all six diterpene alcohol substrates tested (Figure 4b,c). By contrast, the reciprocal CYP71Z25:F86S variant showed only trace product levels, again comparable with the multi‐residue variant of CYP71Z25. Most notably, this protein variant showed substantial activity in producing **19**, making up 24 ± 0.6% of the product profile. Analysis of additional single‐residue variants revealed that S347 hydrogen‐bonds with the docked hydroxy substrates and is conserved in CYP71Z25–CYP71Z29 (Figure 4a,d). The substitution of Thr for S347 impaired enzyme activity in CYP71Z27 except for the conversion of 8,13‐CPP‐derived diterpene alcohols, whereas the same mutation had a lesser impact on CYP71Z25 catalysis, predominantly reducing the conversion of **15** by 2.6% and **24** by 6.4% (Figure 4b). Consistent with the observed protein variant activities, the docking of the **15**‐derived substrate into the active site crevice of CYP71Z25 and the corresponding S347T variant suggests that the exchange of the native S347 residue to Thr results in hydrogen bond formation with a neighboring Pro residue (P413), rather than the hydrogen bond formed with the substrate in the wild‐type enzymes (Figure 4d). Reciprocal exchange of residues E/D283 significantly reduced product formation in CYP71Z25, especially with regards to the conversion of **15**, **23** and **24** (Figure 4b,e), whereas the corresponding variant CYP71Z27:D283E showed only minor product changes, with **19** as an additional product contributing 7.7 ± 0.1% to the profile.

Mutagenesis of the remaining selected residues in CYP71Z25 and CYP71Z27, namely N/D95, T/I288, S/T346 and M/I463, showed limited impact on P450 catalytic specificity, including the formation of **19** as a product by the CYP71Z27:D95N and CYP71Z27:T346S variants, absent in the‐wild type enzyme. Furthermore, the CYP71Z25:T288I variant and an additional double mutation, CYP71Z25:N95D/S346T, showed a 9% increase in producing **28** when co‐expressed with the *trans‐cis*‐CLPP synthase *Sd*CPS2 (Figure S4).

### P450‐derived furanoditerpenoids are present in switchgrass root tissue

**Figure 5:**
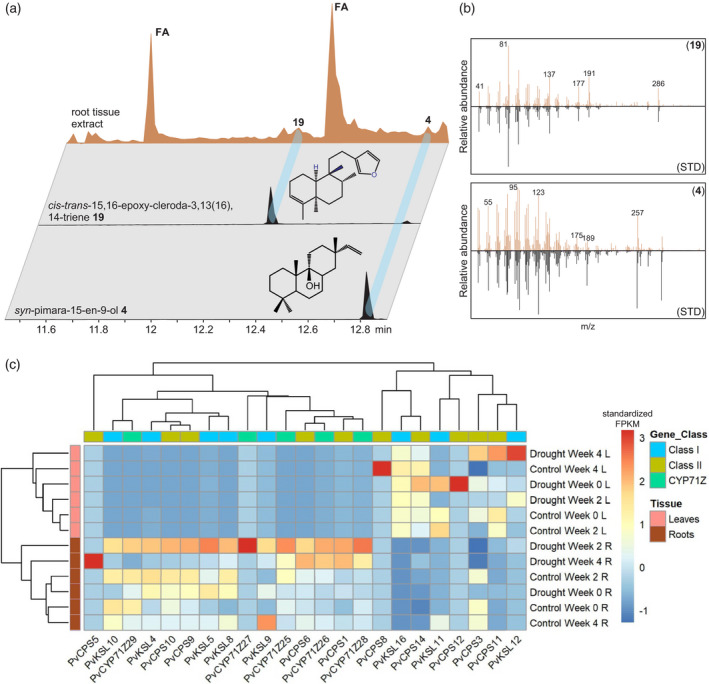
GC‐MS total ion chromatograms (a) and mass spectra (b) of diterpenoids detected in organic solvent extracts of switchgrass roots identified diterpenoids **4** and **19**. FA, fatty acid derivative. (c) Hierarchical cluster analysis of select *diTPS* and *CYP71Z25–CYP71Z29* gene expression profiles from drought‐stressed tissues. Samples were collected before starting treatment (W0), and then after 2 weeks (W2) and 4 weeks (W4) of drought stress treatment. Key: C, well‐watered; D, drought stressed; L, leaves; R, roots.

Previous work demonstrated that select diTPS transcripts and corresponding enzyme products, including **3** and **4**, accumulate in switchgrass (Alamo) leaves and roots exposed to below‐ground oxidative stress (Pelot et al., 2018). To determine whether furanoditerpenoid biosynthesis follows similar patterns, we examined the abundance of diterpenoids and corresponding biosynthetic genes in switchgrass plants exposed to 4 weeks of drought stress or well‐watered conditions. It should be noted that, with the use of nutrient‐enriched water for optimal switchgrass cultivation, additional stress symptoms resulting from nutrient deficiency in drought‐treated plants cannot be excluded. Targeted GC‐MS metabolite profiling of organic solvent root extracts of drought‐stressed and well‐watered switchgrass plants illustrated, albeit at low abundance, the presence of the diTPS product *syn*‐pimara‐15‐en‐9‐ol (**4**) and the furanoditerpenoid *cis‐trans*‐15,16‐epoxy‐cleroda‐3,13(16),14‐triene (**19**), as verified by comparison with the authentic enzyme products (Figure 5a,b). However, the diterpenoid compounds detected did not show a significant accumulation in drought‐stressed plants, as compared with well‐watered control plants. Differential gene expression analysis using the same tissue samples was used to assess *diTPS* and *P450* gene expression patterns in drought‐stressed and control leaf and root tissue (Figure 5c). Gene expression of *CYP71Z25–CYP71Z29* and the majority of diterpenoid metabolic diTPSs, including the *cis‐trans*‐CLPP synthase *PvCPS1*, the predicted *syn*‐CPP synthases *PvCPS9* and *PvCPS10*, and the downstream‐acting class I *diTPS*s *PvKSL4*, *PvKSL5* and *PvKSL8*, was detected only in roots, consistent with the presence of compounds **4** and **19**. By contrast, the 8,13‐CPP (**10**) synthase *PvCPS3* was only moderately expressed in both organs, whereas the *ent*‐LPP (**8**) synthase *PvCPS11* and the putatively gibberellin‐biosynthetic *ent*‐CPP (**7**) synthase *PvCPS14* were expressed only in leaves. Distinct from its homologs, *PvCPS9* and *PvCPS10*, the *syn*‐CPP (**9**) synthase *PvCPS8* was expressed only in leaves, with the highest expression after 4 weeks of treatment in well‐watered plants, suggesting an expression profile at only later developmental stages. *CYP71Z25–CYP71Z29*, as well as all tested *diTPS* genes, showed moderately increased gene expression levels as compared with well‐watered samples, with the highest expression levels observed after 2 weeks of drought treatment, followed by a decrease in transcript abundance at 4 weeks of treatment (Figure 5c). In leaves, the expression of the specialized class II *diTPS*s *PvCPS3* and *PvCPS11* was highest after 4 weeks of drought treatment, whereas *PvCPS8* showed no drought‐elicited transcript accumulation.

Considering a possible role of phosphatases in producing the primary diterpene alcohol substrates of CYP71Z25–CYP71Z29, we further examined the co‐expression of predicted phosphatase genes in the switchgrass genome. Indeed, mutual rank (MR)‐based analysis showed significant co‐expression of *CYP71Z25* and *CYP71Z28* with a putative dolichyldiphosphatase (Pavir.1KG467700) (Data S1). In addition, several putative *Nudix hydrolase* genes, shown to have roles in plant terpenoid dephosphorylation (Henry et al., 2018), showed comparable co‐expression patterns to *CYP71Z25–CYP71Z29* and switchgrass *diTPS* in drought‐stressed root tissue (Figure S11).

## DISCUSSION

Molecular crop improvement strategies require knowledge of the gene–metabolite relationships that contribute to desired crop traits and serve as resources for crop engineering or breeding (Ding et al., 2021; Jez et al., 2016). In particular, an understanding of the dynamic networks of plant specialized metabolites that enable plants to adapt to their environmental niche is needed to address the worsening crop losses caused by climate shifts and associated pest and disease damage (Savary et al., 2019). Diterpenoids serve as key components of biotic and abiotic stress resilience in rice and maize (Kitaoka et al., 2015a,b; Murphy and Zerbe, 2020; Schmelz et al., 2014), and stress‐inducible, species‐specific diterpenoid networks have also been discovered in other food and bioenergy crops, such as foxtail millet, switchgrass and wheat, although their physiological functions are less well understood (Ding et al., 2019; Karunanithi et al., 2020; Pelot et al., 2018; Schmelz et al., 2014; Wu et al., 2012; Zhou et al., 2012). Prior studies identified an expansive *diTPS* gene family in allotetraploid switchgrass (*Panicum virgatum*) that forms specialized diterpenoids both common among the grass family and, to current knowledge, uniquely present in switchgrass (Pelot et al., 2018). The discovery of a group of functional *P450* genes, *CYP71Z25–CYP71Z29*, that catalyze the heterocyclization of several diterpene alcohol substrates derived from class II diTPS products provides a deeper understanding of the divergence of plant diterpenoid metabolism and how the natural modularity of diterpenoid biosynthetic pathways drives the evolution of complex, lineage‐specific blends of bioactive metabolites.

The identification of CYP71Z25–CYP71Z29 in switchgrass, as compared with only two possible paralogs in the diploid switchgrass relative *P. hallii*, suggests an expansion of this P450 group after the split from *P. hallii* approximately 8 MYA (Lovell et al., 2021). The phylogenetic separation of CYP71Z25–CYP71Z29 from other monocot CYP71Z‐type P450s indicates a shared evolutionary origin. The localization of *CYP71Z26* and *CYP71Z27* on chromosome 1K, along with a near‐identical gene arrangement of two paralogs in *P. hallii*, whereas *CYP71Z25*, *CYP71Z28* and *CYP71Z29* are located on subgenome K, suggests that gene family expansion was associated with switchgrass subgenome expansion approximately 4.6 MYA (Lovell et al., 2021).

**Figure 6:**
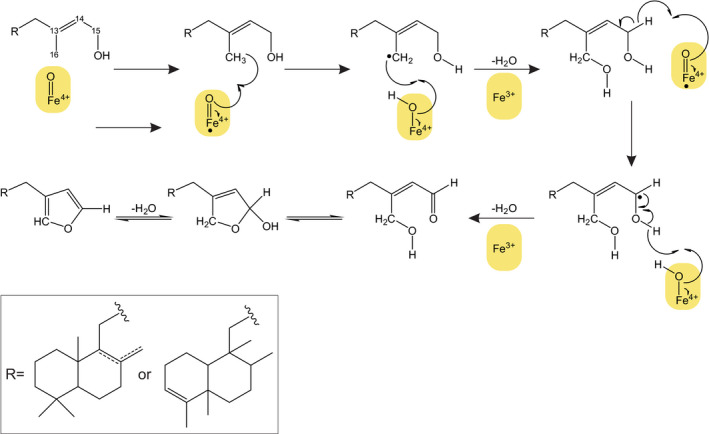
Proposed mechanism for the addition of a furan ring to switchgrass diterpenoid scaffolds, catalyzed by CYP71Z25–CYP71Z29, that may proceed through radical‐based allylic oxidation at C16, followed by aldehyde formation through radical‐based oxidation and final cyclization and water elimination to yield the furan ring.

Diterpenoid‐forming members of the CYP71Z subfamily also exist in maize and rice, where they catalyze position‐specific hydroxylation, carboxylation or epoxidation reactions in various labdane scaffolds produced by the pairwise activity of class II and class I diTPS enzymes (Ding et al., 2019; Mafu et al., 2018; Wu et al., 2011). Consistent with their phylogenetic distance from maize and rice CYP71Z enzymes and a protein sequence identity of less than 75%, the functional characterization of switchgrass CYP71Z25–CYP71Z29 demonstrated rare P450 activity in catalyzing the addition of a furan ring to the labdane backbone. Known examples of P450‐mediated heterocyclization include the biosynthesis of the monoterpenoid menthofuran catalyzed by CYP71A32 in the *Mentha* genus (Bertea et al., 2001) and the recently demonstrated formation of dihydro‐furan intermediates in *Salvia miltiorrhiza* tanshinone biosynthesis catalyzed by members of the CYP71D subfamily (Ma et al., 2021). Similar to these CYP71D enzymes, the catalytic reactions of CYP71Z25–CYP71Z29 may proceed via a typical P450‐mediated, radical‐based allylic oxidation at C16, followed by aldehyde formation through radical‐based oxidation and subsequent cyclization and water elimination to yield the furan ring structure (Figure 6). Co‐expression of CYP71Z25–CYP71Z29 with select class II diTPSs and substrate feeding assays support that class II diTPS‐derived diterpene alcohols, rather than the corresponding 15‐pyrophosphate compounds, are the preferred CYP71Z25–CYP71Z29 substrates. Formation of the 15‐hydroxylated diterpene substrate could be facilitated by a class I diTPS, with a similar function shown in the biosynthesis of labda‐7,13*E*‐dien‐15‐ol in *Selaginella moellendorffii* (Mafu et al., 2011). No such function has yet been demonstrated in switchgrass (Muchlinski et al., 2019; Pelot et al., 2018), but may be encoded by a yet uncharacterized member of the switchgrass class I TPS family. Alternatively, the 15‐hydroxy diterpene intermediate may derive from phosphatase‐mediated dephosphorylation of the class II diTPS products, thus bypassing the common pairwise activity of class II and class I diTPSs. Genomic clustering of *CYP71Z25* and *CYP71Z26/27* with the class II diTPSs *PvCPS2* and *PvCPS1*, but with no class I diTPSs (Figure 1a), and co‐expression of *CYP71Z25–CYP71Z29* with *PvCPS1* and other select class II *diTPS*s in drought‐stressed switchgrass roots support this hypothesis. Although patterns of co‐expression of *CYP71Z25–CYP71Z29* with a predicted *dolichyldiphosphatase* and select *Nudix hydrolases*, which could possibly catalyze the dephosphorylation of class II diTPS products, was also observed in switchgrass roots (Figure S11), experimental evidence for the involvement of phosphatases in labdane diterpenoid metabolism is presently lacking.

The primary formation of class I diTPS products rather than furanoditerpenoids when co‐expressing class II diTPSs, class I diTPSs and CYP71Z25–CYP71Z29 may suggest that the combined activity of class II and class I diTPSs represents the dominant pathway route. Compartmentalization of plastidial class II and class I diTPSs and common P450 localization at the endoplasmic reticulum supports pairwise diTPS activity as the primary pathway. However, the presence of the P450‐derived furanoditerpenoid **19** alongside the class I diTPS product **4** in switchgrass roots suggests the co‐occurrence of both pathway branches *in planta*. The co‐expression of *PvCPS1* and *CYP71Z25–CYP71Z29*, as well as *PvKSL4* and the predicted *syn*‐CPP synthases *PvCPS9* and *PvCPS10* in switchgrass roots is consistent with this hypothesis (Figure 5c). The expression of the *syn*‐CPP synthase *PvCPS8* only in leaves may indicate that the predicted functional homologs *PvCPS9* and/or *PvCPS10* are more likely to serve in the biosynthesis of **17** and **4** in roots. Given the large size of the switchgrass diTPS family and the catalytic overlap of CYP71Z25–CYP71Z29, future biochemical and genetic studies will be required to precisely decode the interactions governing the modular formation of switchgrass‐specific diterpenoids. Interestingly, the lack of CYP71Z25–CYP71Z29 activity with substrates of *ent*‐stereochemistry (including the GA precursor *ent*‐CPP) demonstrates a dedicated role of this P450 group in specialized metabolism and highlights a biochemical separation of furanoditerpenoid and general diterpenoid metabolism in switchgrass. Similar impacts of substrate specificity on partitioning different diterpenoid branches have recently been described in maize, where the specificity of two class I diTPSs, *Zm*KSL2 and *Zm*KSL4, for producing distinct positional isomers of *ent*‐kaurene contributes to the partitioning of pathways towards dolabralexin and kauralexin diterpenoids (Ding et al., 2019).

The presence of specialized diterpenoids (Figure 5) (Pelot et al., 2018) in switchgrass roots expands our knowledge of species‐specific diterpenoid formation in roots, as demonstrated for the stress‐elicited accumulation of diterpenoids with allelopathic and antimicrobial bioactivities in rice roots (Schmelz et al., 2014), and the accumulation of diterpenoid dolabralexins and sesquiterpenoid zealexins in response to drought, oxidative and salinity stress in maize (Christensen et al., 2018; Mafu et al., 2018; Murphy and Zerbe, 2020; Schmelz et al., 2014; Vaughan et al., 2015). Although no furanoditerpenoid accumulation was observed in response to drought stress, the moderately increased transcript abundance of *CYP71Z25–CYP71Z29* and the majority of analyzed *diTPS*s may point to a role in drought or broader abiotic stress defenses in switchgrass roots. Given the structural distinctiveness of switchgrass diterpenoids, it is conceivable that switchgrass furanoditerpenoids exert different bioactivities in interorganismal and environmental interactions. It also appears likely that the switchgrass furanoditerpenoids detected represent lower abundant pathway intermediates that undergo further functional decorations to generate bioactive pathway end products. Indeed, among the approximately 400 known furanoditerpenoids broadly distributed across the plant kingdom, the vast majority feature extensive modifications of both the furan ring and the diterpene skeleton that may include variations of hydroxylation, carboxylation, lactonization, glycosylation and other transformations (Bao et al., 2016).

Integrating sequence comparison, molecular dynamics analysis and site‐directed mutagenesis proved a powerful tool for identifying active site residues contributing to CYP71Z25–CYP71Z29 catalysis and substrate specificity. The relative ease by which the substrate specificity of CYP71Z25 and CYP71Z27 for labdane intermediates of different stereochemistry and double bond configuration could be altered by minor active site modifications supports rapid P450 functional divergence during the evolution of switchgrass diterpenoid metabolism. This is exemplified by a near‐complete functional conversion of CYP71Z27 to the product profile of CYP71Z25 with a single S86 → F substitution. However, the loss of activity in the reciprocal CYP71Z25 variant suggests that different active site positions play major roles in product specificity among the P450s identified. Indeed, the proximity of the F86 aromatic ring to the docked substrate in CYP71Z27 supports a role in substrate orientation in the active site crevice, whereas the introduction of a bulky Phe side chain in the CYP71Z25 cavity may lead to steric hindrance of substrate binding and catalysis (Figure 3c). Further mechanistic insight was gained from the substitution of a conserved Ser in the SRS5 domain shown to be imperative for catalysis by hydrogen bonding to the substrate. Molecular dynamics results suggest that the substitution of this position for Thr leads to reduced conformational flexibility and associated side‐chain rotations that are critical for hydrogen bond formation with the substrate. Paired with the substrate promiscuity of CYP71Z25–CYP71Z29, these mechanistic insights provide a foundation for producing a broader range of furanoditerpenoid natural products. Notably, many plant furanoditerpenoids have been associated with therapeutic bioactivities, ranging from anti‐allergic and anti‐diabetic to anti‐cancer and anti‐viral efficacies, whereby the furan group serves as a key pharmacophore (Bao et al., 2016). In this context, the potential of combinatorial pathway reconstruction for diterpenoid manufacturing is highlighted by the capacity of CYP71Z25 and CYP71Z26 to form the corresponding di‐hydroxy and furan derivatives of **25**, the precursor in the biosynthesis of salvinorin A (Pelot et al., 2017), which is a natural product of *S*. *divinorum* that was identified as a drug candidate for the treatment of drug addictions through its agonistic activity on brain kappa‐opioid receptors (Kivell et al., 2014). The increased catalytic specificity observed in two CYP71Z25 variants further underscores the potential of structure‐guided protein engineering for enabling the desired P450 activities as more mechanistic insight into diTPS and P450 functions is gained.

## EXPERIMENTAL PROCEDURES

### Gene synthesis

CYP71Z25–CYP71Z29 were codon‐optimized for expression in *E. coli* (Kitaoka et al., 2015b) and synthesized as N‐terminally modified genes by the replacement of the sequence upstream of the LPP motif with the leader sequence MAKKTSSKGK (Swaminathan et al., 2009) (Table S2) and individually inserted into MCS2 of a pETDuet‐1 vector (Merck, https://www.merckmillipore.com) carrying the full‐length, codon‐optimized maize cytochrome P450 reductase (*Zm*CPR2) in MCS1. Gene synthesis and cloning were performed by the DOE Joint Genome Institute (JGI) with support through a DNA Synthesis Award (#2568). In addition, synthesized multi‐residue variants of CYP71Z25 and CYP71Z27 were obtained from GenScript® (https://www.genscript.com).

### *Escherichia coli* co‐expression assays

The co‐expression of diTPSs and P450s was conducted on an *E. coli* platform engineered for diterpenoid production (Cyr et al., 2007; Morrone et al., 2010; Murphy et al., 2019). In brief, pIRS and pGGxC plasmids (Morrone et al., 2010) carrying class II diTPSs with distinct products – *ent*‐CPP **7** (*Z. mays* AN2; Harris et al., 2005; used here in place of the native *Pv*CPS14 because of its higher catalytic activity), *syn*‐CPP **9** (*O. sativa* CPS4; Xu et al., 2004; used here in place of the native *Pv*CPS8 because of its higher catalytic activity), (+)‐CPP **20** (*Abies grandis* abietadiene synthase variant D621A; Peters et al., 2001), 8,13‐CPP **10** (*Pv*CPS3), 7,13‐CPP **21** (*Grindelia robusta* 7,13‐CPP synthase; Zerbe et al., 2015), *ent‐*LPP **8** (*Pv*CPS11), *cis‐trans*‐CLPP **11** (*Pv*CPS1) and *trans‐cis*‐CLPP **22** (*Salvia divinorum* CPS2; Pelot et al., 2016) – were co‐expressed with different P450 genes. For the additional co‐expression of class I diTPSs, respective genes subcloned into the pET28b(+) expression vector were used (Pelot et al., 2018). Constructs were co‐transformed into *E. coli* BL21DE3‐C43 cells (Lucigen, https://www.lucigen.com) and cultures were grown at 37°C and 200 rpm in 50 ml of Terrific Broth (TB) medium to an OD_600_ of approximately 0.5–0.6 before cooling to 16°C, and induction with 1 mM isopropyl‐β‐d‐1‐thiogalacto‐pyranoside (IPTG) and the addition of 40 mM sodium pyruvate, 1 mM MgCl_2_, 5 mg L^−1^ riboflavin and 75 mg L^−1^ 5‐aminolevulinic acid (Murphy et al., 2019). After 72 h of incubation, metabolites were extracted with hexane and air‐dried for GC‐MS analysis on an Agilent 7890B GC (Agilent, https://www.agilent.com) interfaced with a 5977 Extractor XL MS Detector at 70 eV and 1.2 ml min^−1^ He flow, using an Agilent DB‐XLB column (30 m, 250 µm i.d., 0.25 µm film) and the following GC parameters: 50°C for 3 min, 15°C min^−1^ to 300°C, hold for 3 min with pulsed split‐less injection at 250°C. MS data from 40–400 mass‐to‐charge ratio (*m*/*z*) were collected after a 13‐min solvent delay. Metabolite quantification (*n* = 3) was based on normalization to the internal standard 1‐eicosene (Sigma‐Aldrich, https://www.sigmaaldrich.com) and OD_600_ at time of induction of protein expression.

### NMR analysis

For NMR analysis, ≥1 mg of diterpene products was enzymatically produced as outlined above and purified by silica chromatography and semi‐preparative HPLC, as previously described (Murphy et al., 2019). Purified compounds were dissolved in deuterated chloroform (CDCl_3_; Sigma‐Aldrich) containing 0.03% (v/v) tetramethylsilane (TMS). The 1D (^1^H and ^13^C) and 2D (HSQC, COSY, HMBC and NOESY) NMR spectra were acquired on a Bruker Avance III 800‐MHz spectrometer (Bruker, https://www.bruker.com) equipped with a 5‐mm CPTCI cryoprobe using Bruker topspin 3.2 and analyzed with mestrenova 11.0.2 (https://mestrelab.com). Chemical shifts were calibrated against known chloroform (^1^H 7.26 and ^13^C 77.0 ppm) signals.

### Homology modeling and molecular docking

Homology models CYP71Z25–CYP71Z29 were generated with rosettacm (Song et al., 2013) using the crystal structure of *Salvia miltiorrhiza* CYP76AH1 (Gu et al., 2019) (PDB‐ID: 5YM3) as a template, and the lowest energy models were selected for docking with rosettadock (Davis and Baker, 2009; Meiler and Baker, 2006). Input native protein structures almost invariably have regions that score poorly, with force fields arising from energetic strain, and minimization protocols commonly lead to increased deviation from the original wild‐type structure, representing stable proteins. To mitigate these limitations, cycles of minimization with combined backbone/sidechain restraints that are Pareto‐optimal with respect to RMSD to the native structure and energetic strain reduction were used to relax the template protein structure (Nivón et al., 2013). The full‐length sequences for all targets and templates were aligned using promals3d (Pei and Grishin, 2014). Each target sequence was, respectively, threaded onto each template and the threaded partial models aligned in a single global frame. Full‐chain models were then generated by Monte Carlo sampling guided by the Rosetta low‐resolution energy function supplemented with distance restraints from the template structures and a penalty for separation in space of residues adjacent in the sequence. Structures were built using a rosetta ‘fold tree’ (Das and Baker, 2008). The global position of each segment was represented in Cartesian space, whereas the residue backbone and side‐chain conformation in each segment were represented in torsion space. Using the aligned target and template sequences, evolutionary constraints were calculated and used for modeling (Thompson and Baker, 2011). A total of 500 homology models were generated for each variant, where the model with the lowest overall protein score (‘total score’) was used for docking each substrate. First, the heme co‐factor was docked into the model, followed by the docking of the substrate variations, i.e. three possible C15 and C16 substitution arrangements for all five substrates tested – hydroxy (C15=OH, C16=H), pyrophosphate (C15=OPP, C16=H) and dihydroxy (C15=OH, C16=OH) – into the heme model complex. The reactive carbon was heavily weighted within rosetta to be constrained to a distance of 2 ± 1 Å. Each docking simulation generated 1000 docked poses that were filtered by ‘high’ constraint (CST) scores, then subsequently by total score (Sc) for the lowest 25% and then by interaction energy (IE) for the remaining lowest 25%.

### Site‐directed mutagenesis

Select point mutants were generated using whole‐plasmid PCR amplification with site‐specific sense and anti‐sense oligonucleotides (Table S3) and Phusion HF Master Mix polymerase (New England Biolabs, https://www.neb.com). *Dpn*1 treatment was applied to remove template plasmids before transformation into DH5α cells for plasmid propagation. All mutants were sequence verified and functionally characterized using E. coli co‐expression assays, as described above.

### *In* *vivo* feeding study

For substrate feeding assays, the constructs pETDuet‐1:*Zm*CPR2/CYP71Z25 and pETDuet‐1:*Zm*CPR2/CYP71Z29 were individually expressed in *E. coli*, as described above. Expression of the base plasmid pETDuet‐1:*Zm*CPR2 was used as a control. At the time of IPTG induction of protein expression, 10 μM of the diterpene alcohol substrates, **15** or **16**, dissolved in 1:1 (v/v) DMSO:MeOH were added to the culture, followed by incubation and metabolite analysis, as outlined above.

### Metabolite profiling of tissue extracts

A total of 4 g of the frozen tissue were ground to a fine powder and metabolites were extracted in 20 ml of hexane with gentle shaking overnight at 4°C. Extracts were then centrifuged at 1200 ***g*** for 2 minutes and the supernatant was transferred into glass vials, air dried and resuspended in 1 ml of hexane. The resuspended samples were purified and fractionated on a silica column with a gradient of ethyl acetate:hexane. Fractions were again air‐dried, resuspended in hexane and analyzed via GC‐MS analysis, as described for *E. coli* co‐expression assays.

### Transcriptome profiling of drought‐elicited switchgrass tissues

Switchgrass plants (var. Alamo AP13) were propagated from tillers to maintain low genetic variation. Plants were cultivated in 9.5‐L pots in a randomized block design under glasshouse conditions to reproductive stage R1, with a 16‐h light/8‐h dark photoperiod and approximately 22°C day/17°C night, prior to drought treatment. Drought stress treatment was applied by withholding water for 4 weeks, compared with control plants receiving daily drip irrigation with nutrient solution. Relative soil water content was measured weekly using a HydroSense II probe (Campbell Scientific, https://www.campbellsci.com). Leaf and root tissue of drought‐stressed and control plants (*n* = 6) were collected weekly and flash‐frozen in liquid nitrogen for later processing. Total RNA was extracted from 100 mg of tissue using a Monarch® Total RNA Miniprep Kit (New England Biolabs) and treated with *DNase I* for genomic DNA removal. Preparation of cDNA libraries and transcriptome sequencing was performed by Novogene Co. Ltd. (https://en.novogene.com). In brief, following RNA integrity analysis and quantitation, cDNA libraries were generated using a NEBNext^®^Ultra™RNA Library Prep Kit (New England Biolabs) and sequenced on an Illumina Novaseq 6000 sequencing platform (Illumina, https://www.illumina.com) generating 40–80 million 150‐bp paired‐end reads per sample. Raw reads were processed using fastqc (Babraham Bioinformatics, https://www.bioinformatics.babraham.ac.uk/projects/fastqc/), and high‐quality reads were aligned to the reference genome (*P. virgatum* var. Alamo AP13 v5.1) using hisat2. Heat maps were generated using the pheatmap package in r 3.6.3 (https://www.cran‐project.org).

### Phylogenetic analysis

A maximum‐likelihood phylogenetic tree of selected P450 protein sequences (Table S4) was generated using the PhyML server (http://www.atgc‐montpellier.fr/phyml/) with four rate substitution categories, LG substitution model, BIONJ starting tree and 1000 bootstrap repetitions (Guindon et al., 2010).

## AUTHOR CONTRIBUTIONS

PZ conceived the original research and oversaw the data analysis. AM and MJ performed most of the experiments and data analyses. KT conducted plant drought stress experiments, the metabolite profiling of plant tissues and transcriptome analysis. JSF and JS performed protein modeling and molecular docking studies. KAP performed NMR structural analyses. LC, DD and YC assisted with site‐directed mutagenesis studies. JTL performed gene synteny studies. AM, MJ and PZ wrote the article, with contributions from all authors. All authors have read and approved the final version for publication.

## CONFLICT OF INTEREST

The authors declare that they have no conflicts of interest associated with this work.

## Supporting information


**Figure S1**. Identification of CYP71Z25–CYP71Z29 paralogs.
**Figure S2**. Diterpenoid metabolic pathways and compounds relevant to this study.
**Figure S3**. Functional characterization of switchgrass CYP71Z25–CYP71Z29.
**Figure S4**. NMR analysis of *syn*‐15,16‐epoxy‐8(17),13(16),14‐triene (**17**).
**Figure S5**. NMR analysis of 15,16‐epoxy‐8,13(16),14‐triene (**18**).
**Figure S6**. NMR analysis of *cis‐trans*‐15,16‐epoxy‐cleroda‐3,13(16),14‐triene (**19**).
**Figure S7**. Functional characterization of switchgrass P450s.
**Figure S8**. NMR analysis of (+)‐15,16‐epoxy‐8(17),13(16),14‐triene (**23**).
**Figure S9**. Substrate specificity of switchgrass CYP71Z25–CYP71Z29.
**Figure S10**. Structural analysis of CYP71Z25–CYP71Z29.
**Figure S11**. Differential gene expression analysis of putative phosphatase genes.

**Table S1**. Average and standard deviation for each interaction energy (IE) value of filtered docking poses for each P450–substrate combination.
**Table S2**. Synthetic genes used in this study.
**Table S3**. Oligonucleotides used in this study.
**Table S4**. Abbreviations and accession numbers for proteins used for phylogenetic studies.

**Data S1**. Mutual rank co‐expression analysis of CYP71Z25–CYP71Z29.

## Data Availability

Nucleotide sequences of the P450 genes and enzymes characterized in this study are available from Phytozome (https://phytozome.jgi.doe.gov): *C* *YP71Z25* (*Pavir*.*1KG341400*), *CYP71Z26* (*Pavir*.*1KG382300*), *CYP71Z27* (*Pavir*.*1KG382400*), *CYP71Z28* (*Pavir*.*1NG304500*) and *CYP71Z29* (*Pavir*.*1NG309700*). Gene identifiers based on *P. virgatum* (var. Alamo AP13) genome (v5.1). The RNA‐seq data have been submitted to the Sequence Read Archive (SRA), accession no. PRJNA644234. All other relevant data can be found within the article and its supporting materials.
