# Supplementary material for: Cytochrome P450‐catalyzed biosynthesis of furanoditerpenoids in the bioenergy crop switchgrass (Panicum virgatum L.)
Source: Plant J. 2021 Sep 24;108(4):1053–68. doi: 10.1111/tpj.15492 (PMC9292899; doi:10.1111/tpj.15492)
Supplement: Supplementary file 1 — Figure S1. Identification of CYP71Z25–CYP71Z29 paralogs. Figure S2. Diterpenoid metabolic pathways and compounds relevant to this study. Figure S3. Functional characterization of switchgrass CYP71Z25–CYP71Z29. Figure S4. NMR analysis of syn‐15,16‐epoxy‐8(17),13(16),14‐triene (17). Figure S5. NMR analysis of 15,16‐epoxy‐8,13(16),14‐triene (18). Figure S6. NMR analysis of cis‐trans‐15,16‐epoxy‐cleroda‐3,13(16),14‐triene (19). Figure S7. Functional characterization of switchgrass P450s. Figure S8. NMR analysis of (+)‐15,16‐epoxy‐8(17),13(16),14‐triene (23). Figure S9. Substrate specificity of switchgrass CYP71Z25–CYP71Z29. Figure S10. Structural analysis of CYP71Z25–CYP71Z29. Figure S11. Differential gene expression analysis of putative phosphatase genes. [file TPJ-108-1053-s003.pdf]

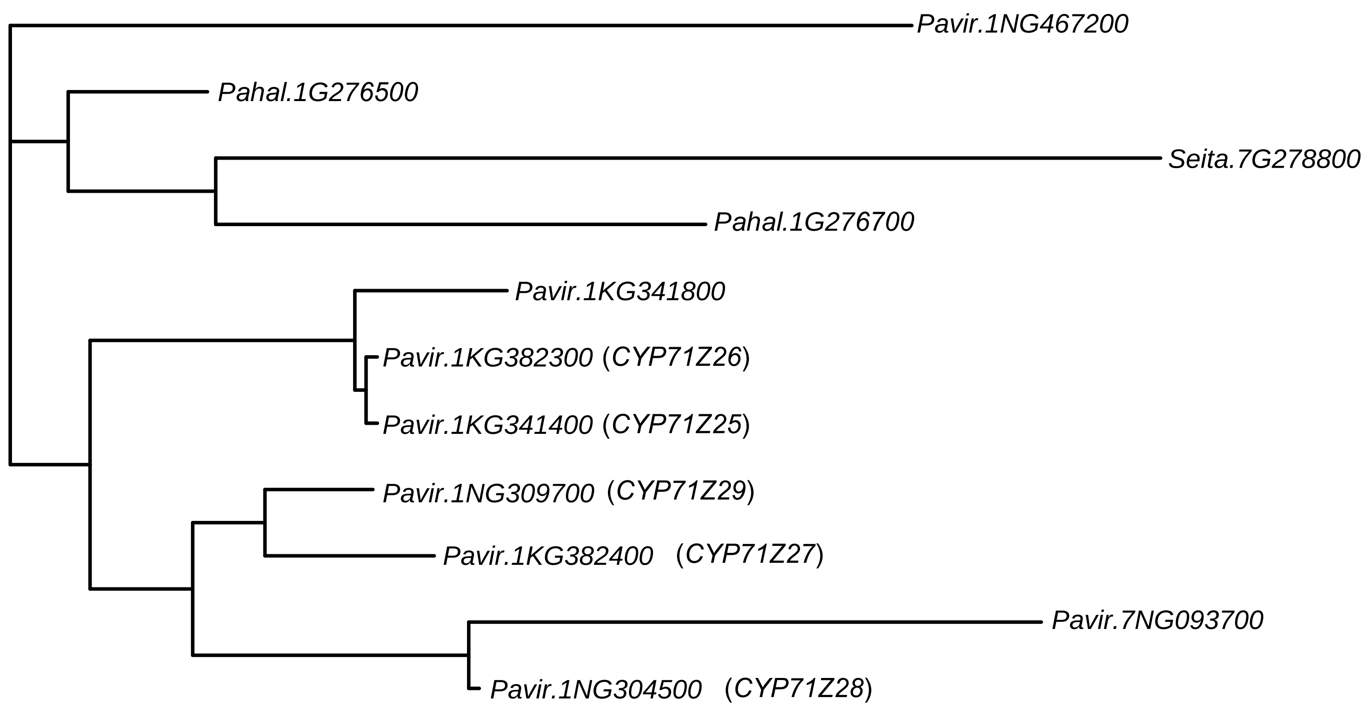

Supplementary Fig. 1: Orthology network of *Panicum virgatum* CYP71Z25-29 paralogs identified in the genomes of *Panicum halli* (v3.1) and *Setaria italica* (v2.2) (https://phytozome.jgi.doe.gov).The genes Pahal.1G276500 and Pahal.1G276700 derived from the unclustered *P. hallii* genome v3.1 are identical to Pahal.A02218 and Pahal.A02220 derived from the cluster *P. halli* genome v2.0 used for analysis of genome localization (Fig. 2).

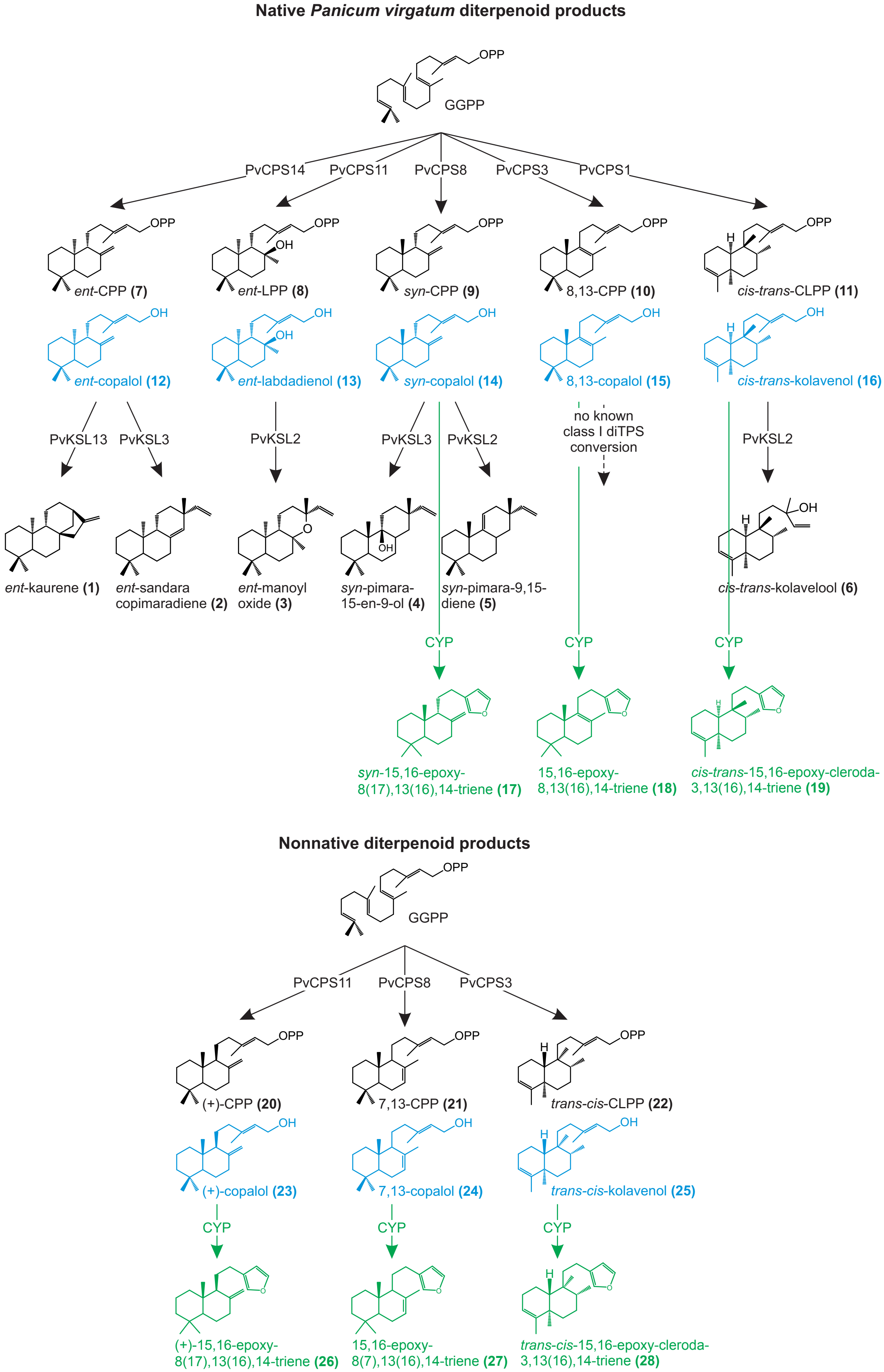

Supplementary Fig. 2. Diterpenoid-metabolic pathways and compounds relevant to this study. Shown are native and nonnative diterpenoid products and corresponding class II and class I diterpene synthases used for the functional analysis of the cytochrome P450 monooxygenases, CYP71Z25-29, in this study. Abbreviations: GGPP, geranylgeranyl pyrophosphate; CPP, copalyl pyrophosphate; LPP, labdadienyl pyrophosphate; CLPP, clerodienyl pyrophosphate; CPS, copalyl diphosphate synthase; KSL, kaurene synthase-like. Blue: alcohol derivatives of corresponding class II diterpene synthase products through the activity of endogenous *E. coli* phosphatases; green: CYP71Z25-29 products from the corresponding class II diterpene synthase alcohol products (12-16, 23-25). Structures of products 26-28 are tentatively assigned based on the structure of the respective class II diterpene synthase products.

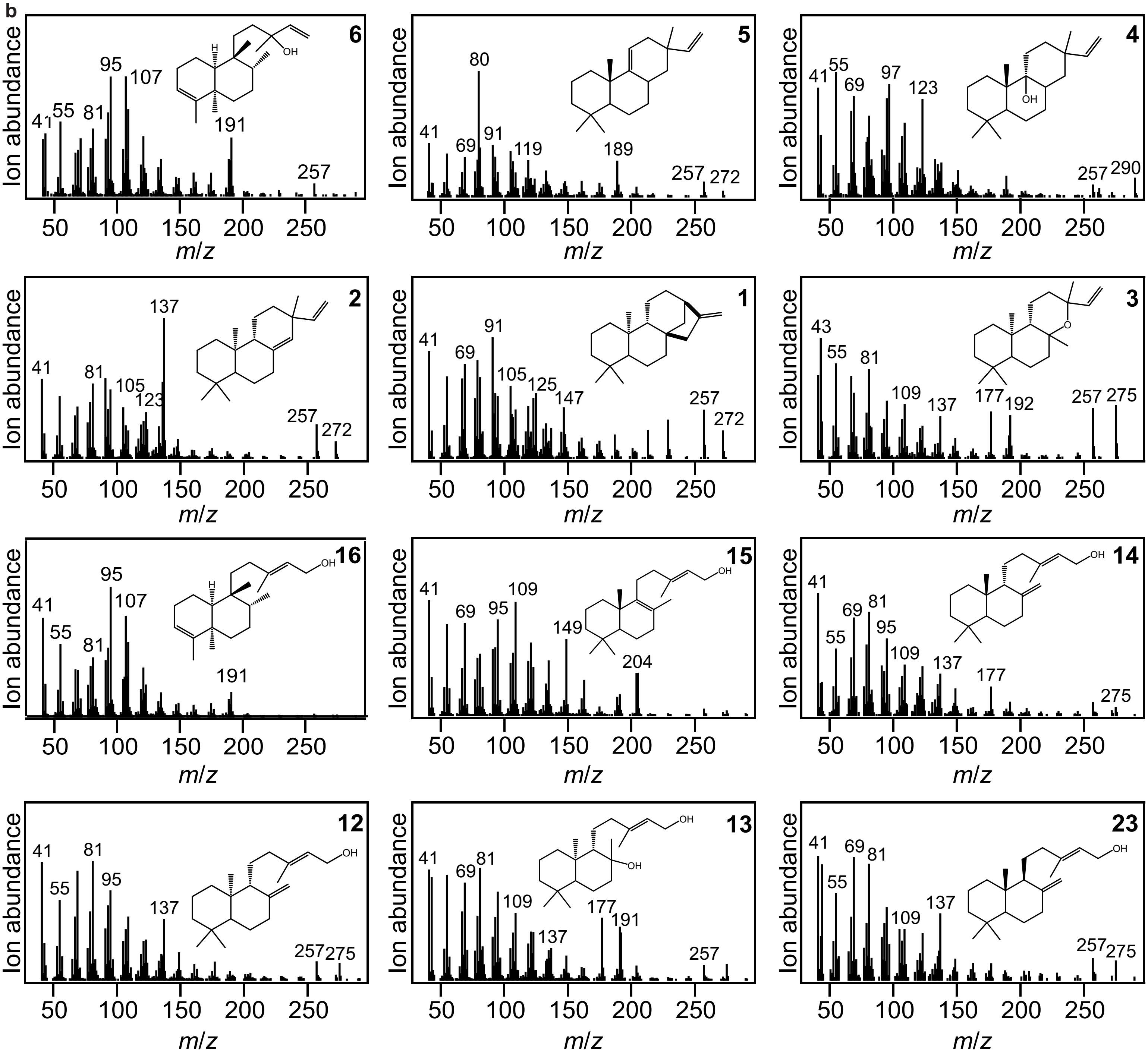

Supplementary Fig. 3. Extracted ion GC-MS mass spectra of relevant products resulting from *E. coli* co-expression of each P450 candidate (CYP71Z25-29) with pairs of diterpene synthases producing core switchgrass diterpene scaffolds. 6, *cis-trans*-kolavelool; 5, *syn*- pimara-9,15-diene; 4, *syn*-pimara-15-ene-9-ol; 2, *ent*-sandaracopimaradiene; 1, *ent*-kaurene; 3, *ent*-manoyl oxide; 16, *cis-trans*-kolavenol (i.e. dephosphorylated *cis-trans*-CLPP); 14, *syn*-copalol (i.e. dephosphorylated *syn*-CPP); 12, *ent*-copalol (i.e. dephosphorylated *ent*-CPP); 13, *ent*-labda-8,15-diol (i.e. dephosphorylated *ent*-LPP); 15, 8,13-copalol (i.e. dephosphorylated 8,13-CPP); 23,(+)-copalol (i.e. dephosphorylated (+)-CPP).

Supplementary Fig 4. NMR analysis of *syn*-15,16-epoxy-8(17),13(16),14-triene (**17**).

Assigned ^{13}C and ^{1}H chemical shifts for **17** (201 MHz and 800 MHz respectively, $CDCl_3$ solvent). The structure for **17** was numbered (A) and determined by the selected 2D NMR NOESY, HMBC and COSY correlations (B).

syn-15,16-epoxy-8(17),13(16),14-triene (**17**)			
Position	δ_C (ppm)	δ_H (ppm)	J (Hz)
1 a	36.74	1.05 (dq)	12.5, 2.8
b		1.54 (m)	
2 a	19.13	1.43 (dt)	13.3, 3.5
b		1.63 (m)	
3 a	42.66	1.15 (td)	13.3, 3.5
b		1.40 (m)	
4	33.23		
5	45.80	1.30 (m)	
6 a	23.64	1.32 (m)	
b		1.63 (m)	
7 a	31.59	2.10 (m)	
b		2.20 (m)	
8	149.08		
9	57.80	1.60 (m)	
10	37.95		
11 a	26.69	1.58 (m)	
b		1.79 (m)	
12 a	23.37	2.14 (m)	
b		2.33 (ddd)	14.6, 10.6, 4.2
13	125.52		
14	111.07	6.27 (br s)	
15	142.56	7.34 (t)	1.7
16	138.73	7.20 (br s)	
17 a	109.63	4.54 (dd)	2.4, 1.7
b		4.73 (t)	2.4
18	33.49	0.87 (s)	
19	22.16	0.81 (s)	
20	22.33	0.92 (s)	

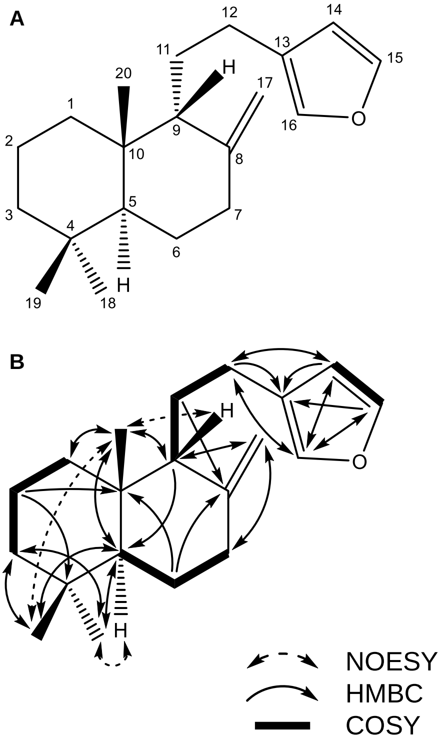

^{13}C NMR spectrum of **17** (201 MHz, $CDCl_3$ solvent).

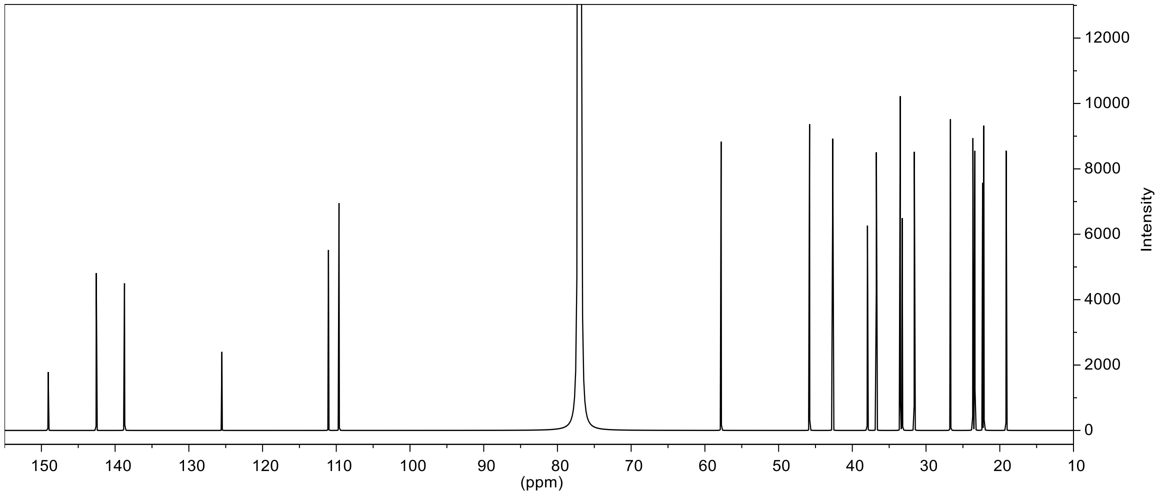

Supplementary Fig. 4, continued

The majority of the bicyclic decalin ^{13}C and ^{1}H NMR chemical shifts of **17** were in agreement (highlighted by *) with the corresponding class II diTPS product of *syn*-copalol (**14**; Yee & Coates (1992) *J. Org. Chem*. 17, 4598-4608).

	(**17**)	(**14**)	(**17**)	(**14**)
Position	δ_C (ppm)	δ_C (ppm)	δ_H (ppm)	δ_H (ppm)
1 a	36.74*	36.76*	1.05 (dq)	1.05 (br d)
b			1.54 (m)*	1.57*
2 a	19.13*	19.15*	1.43 (dt)*	1.45*
b			1.63 (m)*	1.61*
3 a	42.66*	42.68*	1.15 (td)*	1.17 (td)*
b			1.40 (m)*	1.38*
4	33.23*	33.21*		
5	45.80*	45.78*	1.30 (m)*	1.26*
6 a	23.64*	23.64*	1.32 (m)*	1.30*
b			1.63 (m)*	1.59*
7 a	31.59*	31.56*	2.10 (m)*	2.06*
b			2.20 (m)	2.17 (br d)
8	149.08*	149.20*		
9	57.80*	57.90*	1.60 (m)	1.50
10	37.95*	38.00*		
11 a	26.69	24.46	1.58 (m)	1.47
b			1.79 (m)	1.62
12 a	23.37	38.15	2.14 (m)	1.74
b			2.33 (ddd)	1.90
13	125.52	140.58		
14	111.07	122.94	6.27 (br s)	5.41 (tq)
15	142.56	59.44	7.34 (t)	4.15 (d)
16	138.73	16.52	7.20 (br s)	1.67
17 a	109.63*	109.44*	4.54 (dd)*	4.51 (dd)*
b			4.73 (t)*	4.69 (t)*
18	33.49*	33.49*	0.87 (s)*	0.87*
19	22.16*	22.15*	0.81 (s)*	0.80*
20	22.33*	22.35*	0.92 (s)	0.91 (d)

^{1}H NMR spectrum of **17** (800 MHz, $CDCl_3$ solvent).

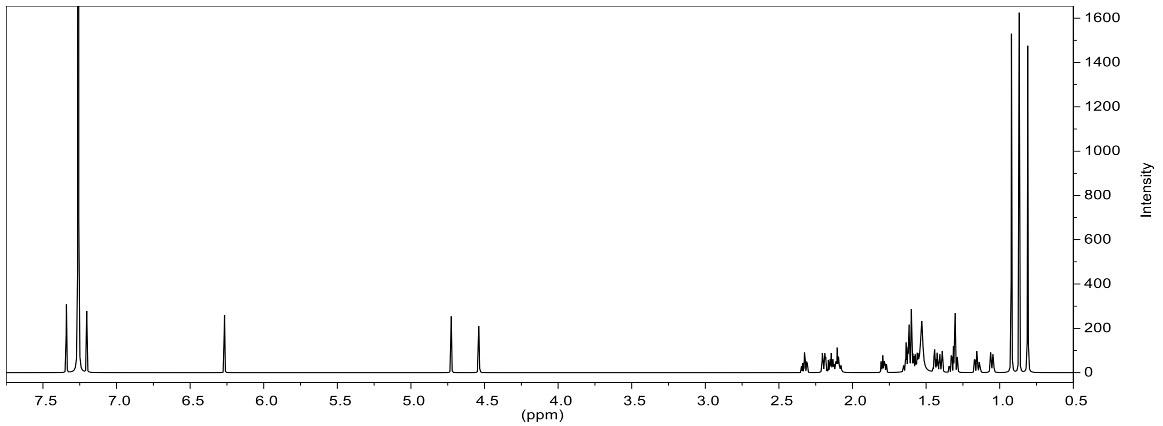

Supplemental Fig 5. NMR analysis of 15,16-epoxy-8,13(16),14-triene (compound **18**).

Assigned ^{13}C and ^{1}H chemical shifts for **18** (201 MHz and 800 MHz respectively, $CDCl_3$ solvent). The structure for **18** was numbered (**A**) and determined by the selected 2D NMR NOESY, HMBC and COSY correlations (**B**).

(15,16-epoxy-8,13(16),14-triene (**18**)			
Position	δ_C (ppm)	δ_H (ppm)	J (Hz)
1 a	37.02	1.21 (td)	12.9, 3.7
b		1.87 (m)	
2 a	19.07	1.50 (dt)	13.7, 4.0
b		1.62 (m)	
3 a	41.81	1.17 (td)	13.7, 4.0
b		1.42 (m)	
4	33.35		
5	51.91	1.15 (dd)	12.7, 2.0
6 a	19.07	1.42 (m)	
b		1.66 (m)	
7 a	33.63	1.96 (dd)	17.6, 6.3
b		2.06 (m)	
8	126.36		
9	140.19		
10	38.99		
11	25.74	2.45 (dt)	11.3, 6.0
12 a	28.76	2.13 (dt)	11.3, 6.0
b		2.27 (td)	12.5, 12.1, 6.0
13	125.70		
14	110.86	6.30 (br s)	
15	142.62	7.35 (t)	1.7
16	138.40	7.23 (br s)	
17	19.56	1.61 (s)	
18	33.33	0.90 (s)	
19	21.71	0.84 (s)	
20	20.09	0.96 (s)	

A

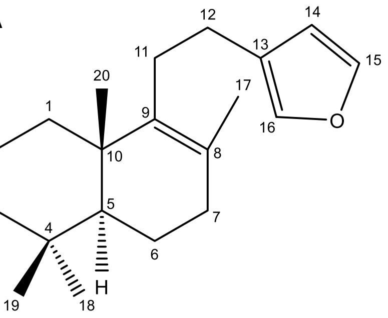

B

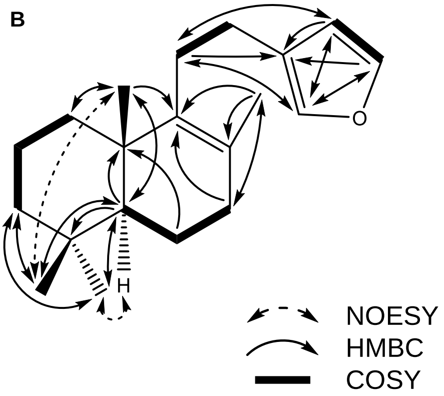

^{13}C NMR spectrum of **18** (201 MHz, $CDCl_3$ solvent).

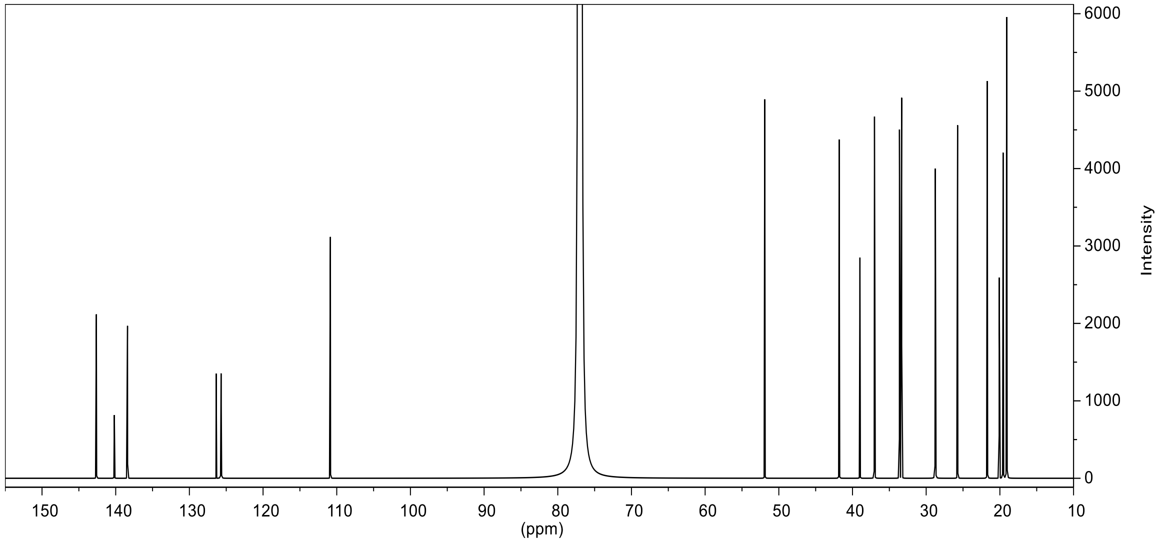

Supplementary Fig. 5, continued

The majority of the bicyclic decalin ^{13}C and ^{1}H NMR chemical shifts of (**18**) were in agreement (highlighted by *) with the corresponding class II diTPS product of 8,13-copalol (**15**; Pelot *et al*. (2018) *Plant Physiol*. 178(1):54-71).

	(**18**)	(**15**)	(**18**)	(**15**)
Position	δ_C (ppm)	δ_C (ppm)	δ_H (ppm)	δ_H (ppm)
1 a	37.02*	36.97*	1.21 (td)	1.16 (m)
b			1.87 (m)	1.82 (dt)
2 a	19.07*	19.07*	1.50 (dt)*	1.48 (dt)*
b			1.62 (m)*	1.59 (m)*
3 a	41.81*	41.80*	1.17 (td)	1.15 (m)
b			1.42 (m)*	1.41 (m)*
4	33.35*	33.33*		
5	51.91*	51.90*	1.15 (dd)*	1.12 (dd)*
6 a	19.07*	19.07*	1.42 (m)*	1.40 (m)*
b			1.66 (m)*	1.64 (dd)*
7 a	33.63*	33.64*	1.96 (dd)*	1.94 (dd)*
b			2.06 (m)*	2.04 (m)*
8	126.36*	126.04*		
9	140.19*	140.13*		
10	38.99*	39.05*		
11 a	25.74	26.77	2.45 (dt)	1.98 (td)
b				2.12 (td)
12 a	28.76	40.16	2.13 (dt)	2.05 (m)
b			2.27 (td)	
13	125.70	140.79		
14	110.86	122.60	6.30 (br s)	5.43 (tq)
15	142.62	59.44	7.35 (t)	4.16 (d)
16	138.40	16.36	7.23 (br s)	1.71 (s)
17	19.56*	19.51*	1.61 (s)*	1.58 (s)*
18	33.33*	33.32*	0.90 (s)*	0.88 (s)*
19	21.71*	21.70*	0.84 (s)*	0.83 (s)*
20	20.09*	20.09*	0.96 (s)*	0.94 (s)*

^{1}H NMR spectrum of **18** (800 MHz, $CDCl_3$ solvent).

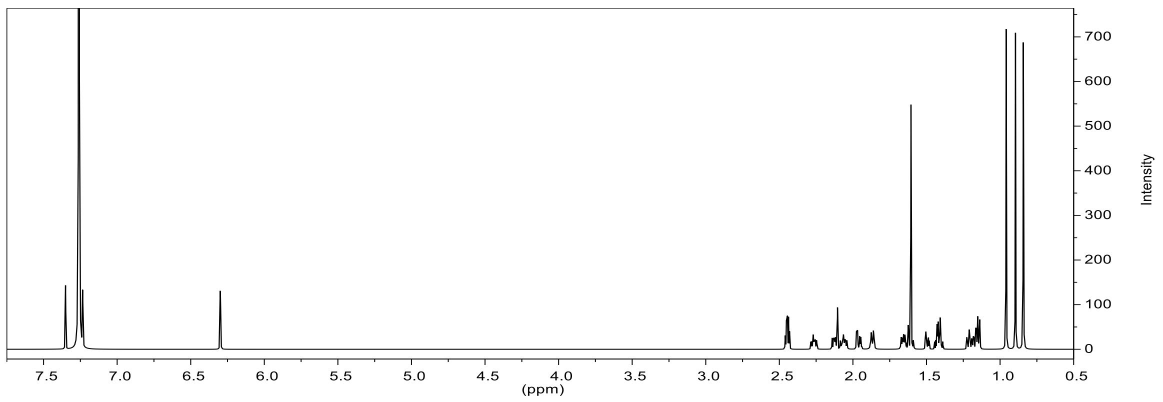

Supplementary Fig. 6. NMR analysis of *cis-trans*-15,16-epoxy-cleroda-3,13(16),14-triene (**19**).

Assigned ^{13}C and ^{1}H chemical shifts for **19** (201 MHz and 800 MHz respectively, $CDCl_3$ solvent). The structure for **19** was numbered (A) and determined by the selected 2D NMR NOESY, HMBC and COSY correlations (B).

cis-trans-15,16-epoxy-cleroda-3,13(16),14-triene (**19**)			
Position	δ_C (ppm)	δ_H (ppm)	J (Hz)
1 a	20.00	1.62 (m)	
b		1.82 (m)	
2 a	25.88	1.94 (m)	
b		2.01 (m)	
3	122.44	5.35 (br s)	
4	142.15		
5	38.74		
6 a	32.31	1.50 (m)	
b		1.63 (m)	
7 a	27.25	1.36 (m)	
b		1.43 (m)	
8	37.43	1.60 (m)	
9	38.61		
10	44.62	1.50 (m)	
11 a	38.25	1.39 (td)	13.6, 4.2
b		1.98 (td)	13.6, 4.7
12 a	18.88	2.33 (td)	13.6, 4.2
b		2.45 (td)	13.6, 4.7
13	126.10		
14	111.06	6.28 (br s)	
15	142.63	7.35 (t)	1.7
16	138.41	7.21 (br s)	
17	15.39	0.88 (d)	7.0
18	19.35	1.65 (q)	1.8
19	27.62	1.18 (s)	
20	26.31	1.08 (s)	

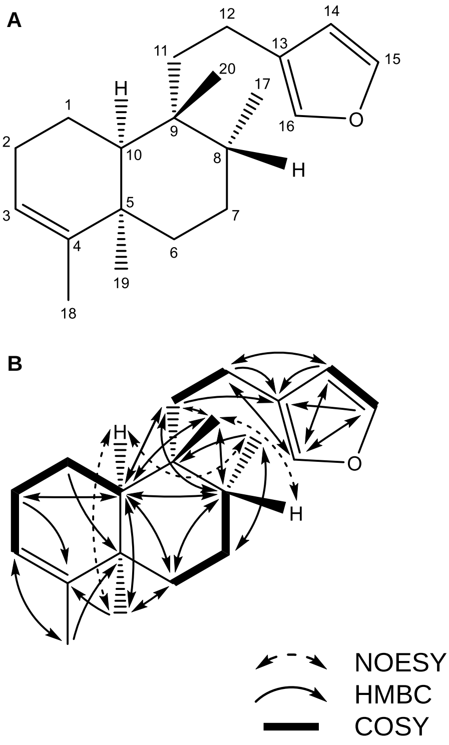

^{13}C NMR spectrum of **19** (201 MHz, $CDCl_3$ solvent).

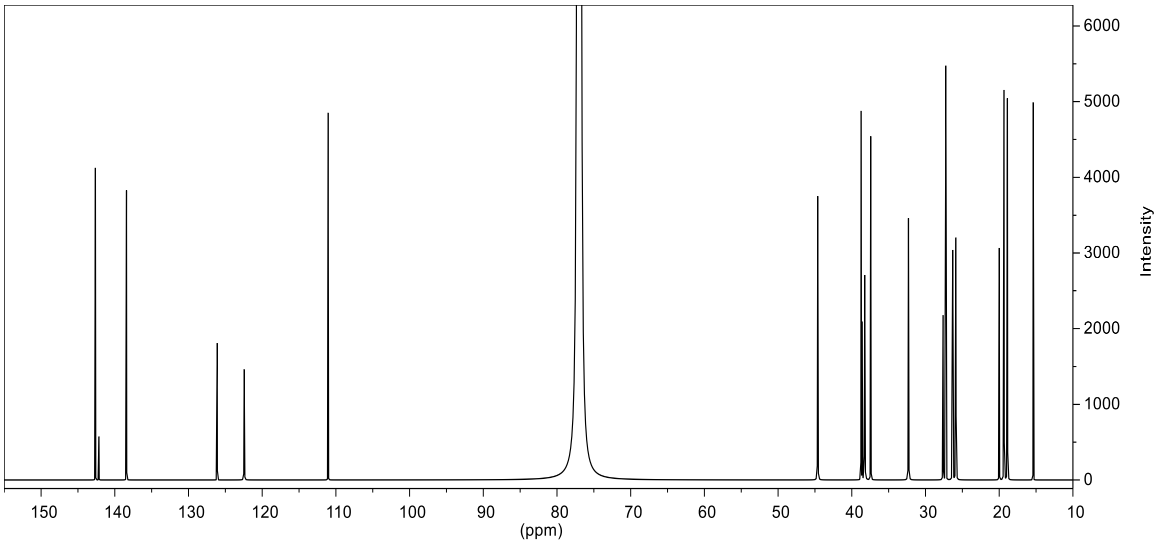

Supplementary Fig. 6, continued

The majority of the bicyclic decalin ^{13}C and ^{1}H NMR chemical shifts of **19** were in agreement (highlighted by *) with the corresponding class II diTPS product of *cis-trans*-kolavenol (**16**; Pelot *et al*. (2018) *Plant Physiol*. 178(1):54-71).

	(**19**)	(**16**)	(**19**)	(**16**)
Position	δ_C (ppm)	δ_C (ppm)	δ_H (ppm)	δ_H (ppm)
1 a	20.00*	20.01*	1.62 (m)*	1.60 (m)*
b			1.82 (m)*	1.80 (m)*
2 a	25.88*	25.87*	1.94 (m)*	1.93 (m)*
b			2.01 (m)*	1.98 (m)*
3	122.44*	122.41*	5.35 (br s)*	5.34 (br s)*
4	142.15*	142.16*		
5	38.74*	38.77*		
6 a	32.31*	32.32*	1.50 (m)*	1.48 (m)*
b			1.63 (m)*	1.61 (m)*
7 a	27.25*	27.25*	1.36 (m)*	1.35 (m)*
b			1.43 (m)*	1.41 (m)*
8	37.43*	37.50*	1.60 (m)*	1.56 (m)*
9	38.61*	38.59*		
10	44.62*	44.63*	1.50 (m)	1.43 (dd)
11 a	38.25	36.03	1.39 (td)	1.23 (td)
b			1.98 (td)	1.83 (td)
12 a	18.88	33.42	2.33 (td)	1.91 (td)
b			2.45 (td)	2.05 (td)
13	126.10	141.35		
14	111.06	122.67	6.28 (br s)	5.42 (tq)
15	142.63	59.48	7.35 (t)	4.16 (d)
16	138.41	16.60	7.21 (br s)	1.70 (br s)
17	15.39*	15.38*	0.88 (d)*	0.86 (d)*
18	19.35*	19.35*	1.65 (q)*	1.64 (q)*
19	27.62*	27.71*	1.18 (s)*	1.15 (s)*
20	26.31*	26.41*	1.08 (s)*	1.02 (s)*

^{1}H NMR spectrum of **19** (800 MHz, $CDCl_3$ solvent).

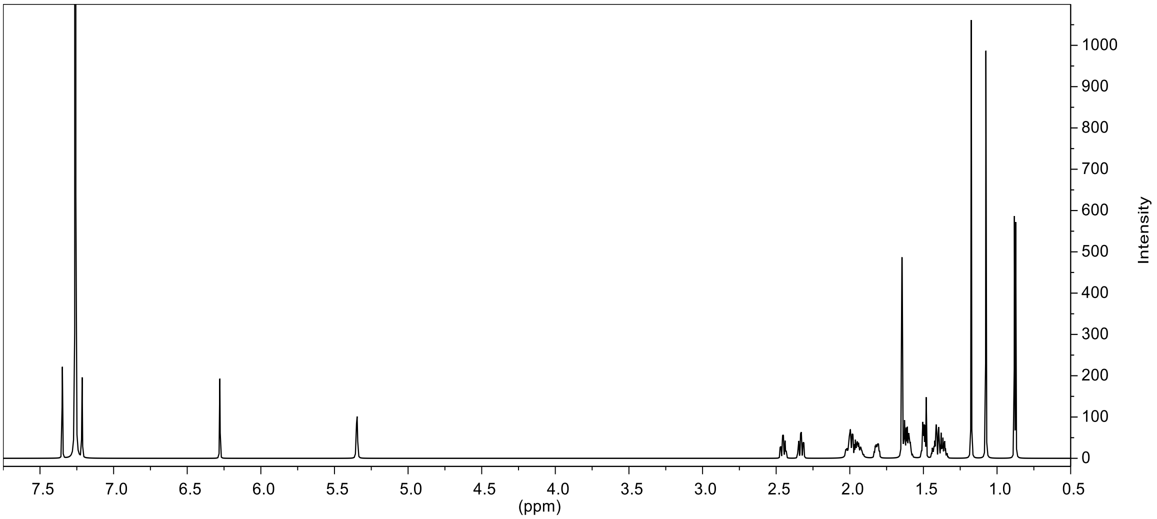

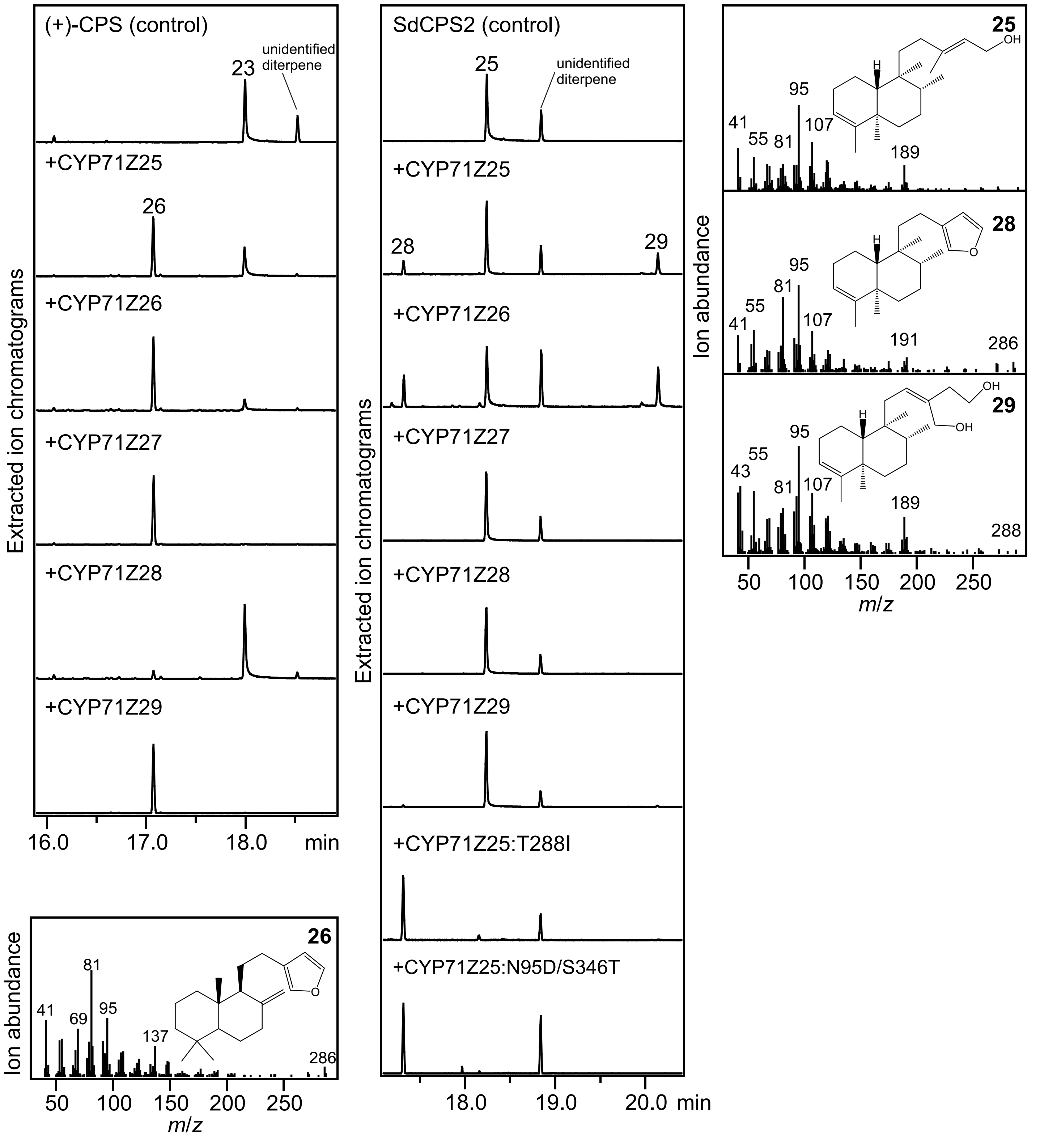

Supplementary Fig. 7. Functional characterization of switchgrass P450s. Individually scaled extracted ion GC-MS chromatograms and corresponding mass spectra of reaction products resulting from *E. coli* co-expression assays of the individual constructs of CYP71Z25 and selected P450 variants with class II diTPSs producing (+)-copalol (i.e. dephosphorylated (+)-CPP) or *trans-cis*-kolavenol (i.e. dephosphorylated *trans-cis*-CLPP produced by *Salvia divinorum* CPS2). 23, (+)-copalol (i.e. dephosphorylated (+)-CPP); 25, *trans-cis*-kolavenol (i.e. dephosphorylated *trans-cis*-CLPP; 26, (+)-15,16-epoxy-8(17),13(16),14-triene; 29, *trans-cis*-cleroda-3,12-dien-15,16-diol; 28, predicted furanoditerpenoid derivative of 25. Structures of the furanoditerpenoid product 28 and dihydroxyl product 29 were assigned on the basis of the respective substrate structures.

Supplementary Fig. 8. NMR analysis of (+)-15,16-epoxy-8(17),13(16),14-triene (**26**).

Assigned ^{13}C and ^{1}H chemical shifts for **26** (201 MHz and 800 MHz respectively, $CDCl_3$ solvent). The structure for **26** was numbered (A) and determined by the selected NMR NOESY, HMBC and COSY correlations (B).

(+)-15,16-epoxy-8(17),13(16),14-triene (**26**)			
Position	δ_C (ppm)	δ_H (ppm)	J (Hz)
1 a	39.02	0.97 (td)	13.0, 3.9
b		1.74 (m)	
2 a	19.39	1.47 (m)	
b		1.56 (m)	
3 a	42.66	1.16 (td)	13.4, 4.1
b		1.39 (m)	
4	33.59		
5	55.47	1.07 (dd)	12.6, 2.8
6 a	24.45	1.33 (m)	
b		1.74 (m)	
7 a	38.35	1.98 (td)	13.0, 5.1
b		2.40 (m)	
8	148.57		
9	56.09	1.64 (m)	
10	39.61		
11 a	24.09	1.61 (m)	
b		1.76 (m)	
12 a	23.62	2.23 (m)	
b		2.55 (m)	
13	125.64		
14	111.00	6.26 (m)	
15	142.63	7.35 (t)	1.6
16	138.68	7.19 (m)	
17 a	106.25	4.56 (m)	
b		4.86 (m)	
18	33.62	0.86 (s)	
19	21.74	0.80 (s)	
20	14.52	0.68 (s)	

A

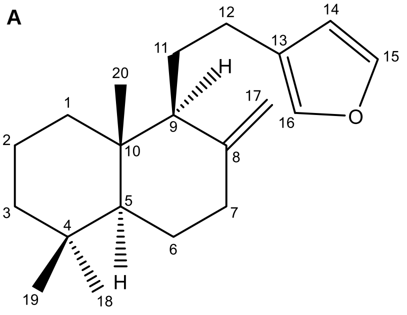

B

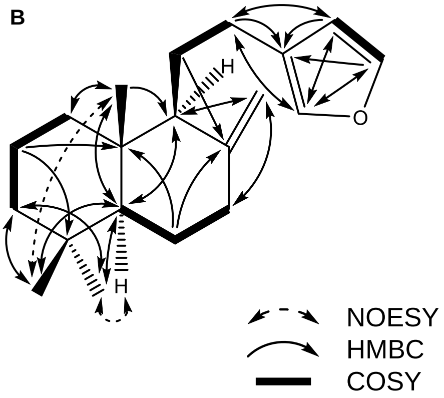

^{13}C NMR spectrum of **26** (201 MHz, $CDCl_3$ solvent).

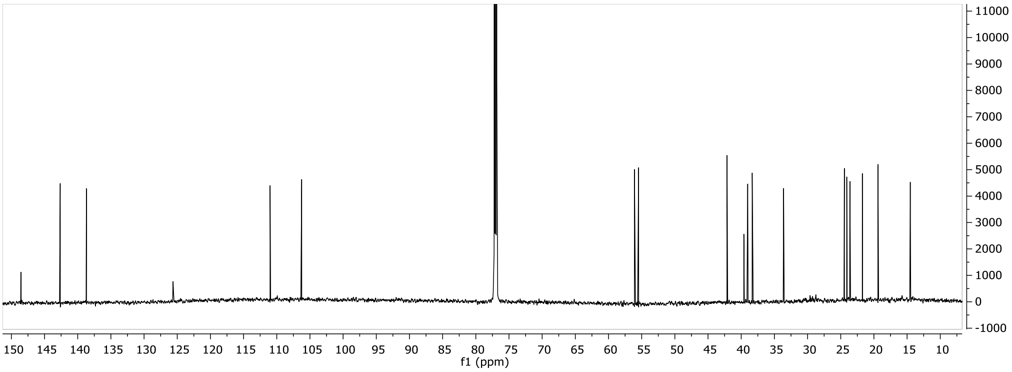

Supplementary Fig. 8, continued

The majority of the bicyclic decalin ^{13}C and ^{1}H NMR chemical shifts of **26** were in agreement (highlighted by *) with the corresponding class II diTPS product of (+)-copalol (**23**; Yee & Coates (1992) *J. Org. Chem*. 17, 4598-4608).

H_2O peak

	(**26**)	(**23**)	(**26**)	(**23**)
Position	δ_C (ppm)	δ_C (ppm)	δ_H (ppm)	δ_H (ppm)
1 a	39.02*	39.19*	0.97 (td) *	0.99 (td) *
b			1.74 (m) *	1.72*
2 a	19.39	21.89	1.47 (m)	1.39*
b			1.56 (m) *	1.57*
3 a	42.66*	42.27*	1.16 (td) *	1.16 (td)*
b			1.39 (m) *	1.37*
4	33.59*	33.71*		
5	55.47*	55.63*	1.07 (dd) *	1.07 (dd)*
6 a	24.45*	24.56*	1.33 (m) *	1.30*
b			1.74 (m) *	1.70*
7 a	38.35*	38.47*	1.98 (td) *	1.96 (td)*
b			2.40 (m) *	2.38*
8	148.57*	148.74*		
9	56.09*	56.40*	1.64 (m)	1.54
10	39.61*	39.77*		
11 a	24.09	19.52	1.61 (m)	1.45
b			1.76 (m)	1.60
12 a	23.62	38.55	2.23 (m)	1.79
b			2.55 (m)	2.15
13	125.64	140.78		
14	111.00	123.05	6.26 (m)	5.38 (tq)
15	142.63	59.54	7.35 (t)	4.15 (d)
16	138.68	16.49	7.19 (m)	1.65
17 a	106.25*	106.38*	4.56 (m) *	4.50 (br d)*
b			4.86 (m) *	4.82 (app q)*
18	33.62*	33.75*	0.86 (s) *	0.86*
19	21.74*	21.86*	0.80 (s) *	0.79*
20	14.52*	14.62*	0.68 (s) *	0.67*

^{1}H NMR spectrum of **26** (800 MHz, $CDCl_3$ solvent). (Note peak 1.55 represents probably water impurity)

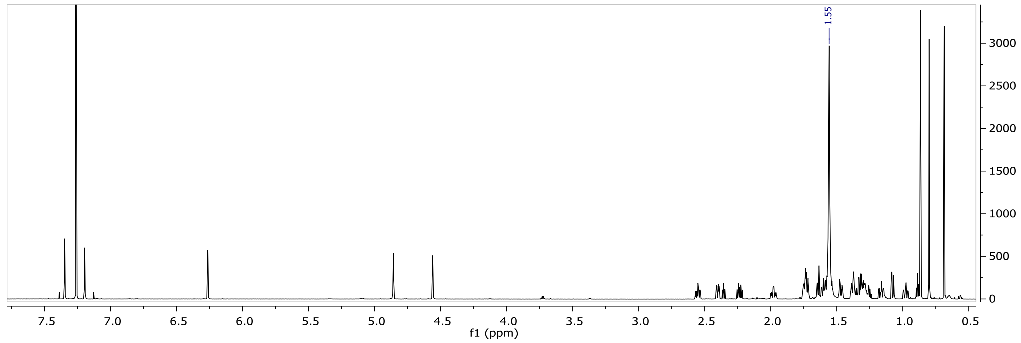

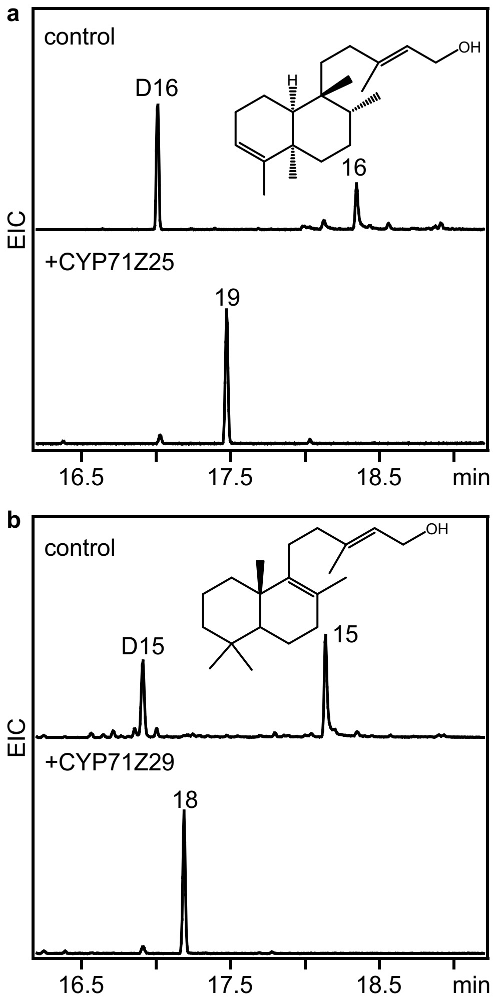

Supplementary Figure 9. Substrate-specificity of switchgrass CYP71Z25-29. Individually scaled extracted ion GC-MS chromatograms of enzyme products resulting from *E. coli* expression of CYP71Z25 or CYP71Z29 with purified diterpenoid alcohol substrates added to the culture. (**a**) *E. coli* expression of CYP71Z25 with purified 16 (dephosphorylated 11) added as a substrate. Compound D16 is a derivative of 16 presumably through isomerization during the *E. coli* expression. (**b**) *E. coli* expression of CYP71Z29 with purified 15 (dephosphorylated 10) added as a substrate. Compound D15 is a putative derivative of 15 presumably through isomerization during the *E. coli* expression.

a b c

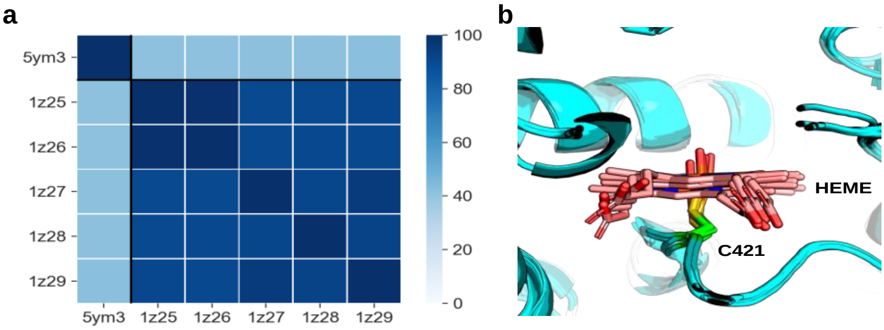

Variant	RMSD
1z25	0.706
1z26	0.675
1z27	0.692
1z28	0.751
1z29	0.680

d e

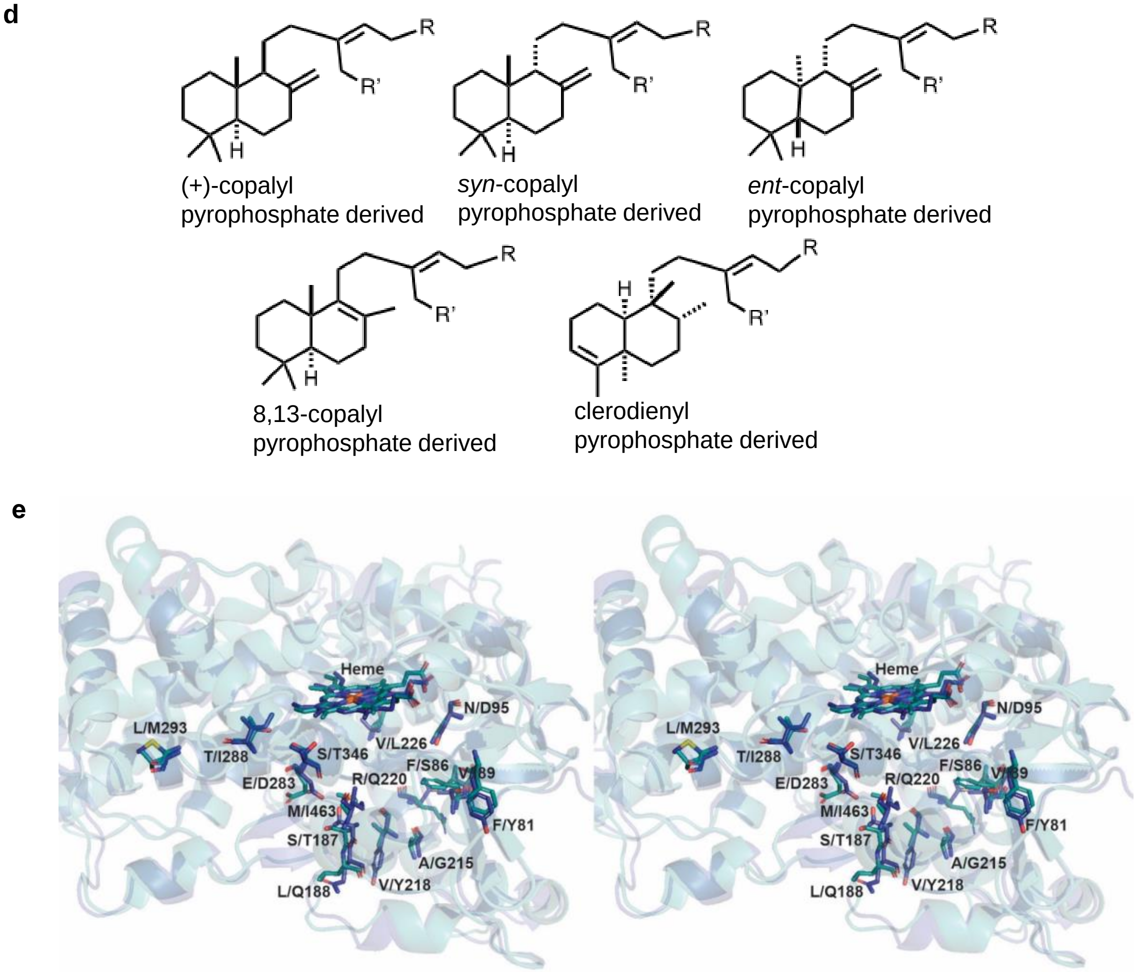

Supplementary Fig. 10: Structural analysis of CYP71Z25-29. (**a**) Heatmap of pairwise identity between the template (*Salvia miltiorrhiza* CYP76AH155; PDB-ID 5YM3) and CYP71Z25-29, and between each P450 variant. (**b**) Overlay of each CYP71Z25-29 models active site (teal, template gray) with heme (pink) and residue C421 (green). (**c**) RMSD (root-mean-square deviation) values of the generated structural models of CYP71Z25-29 as compared to the template (**d**) Structures of the three possible substitution arrangements (15-hydroxy, R=OH, R'=H; 15-pyrophosphate, R=OPP, R'=H; 15,16-dihydroxy, R=OH, R'=OH for all five P450 substrates investigated via ligand docking in this study. (**e**) Stereo view representation of a structural overlay of homology models of CYP71Z25 (teal) and CYP71Z29 (blue), illustrating active site residues targeted for site-directed mutagenesis in this study.

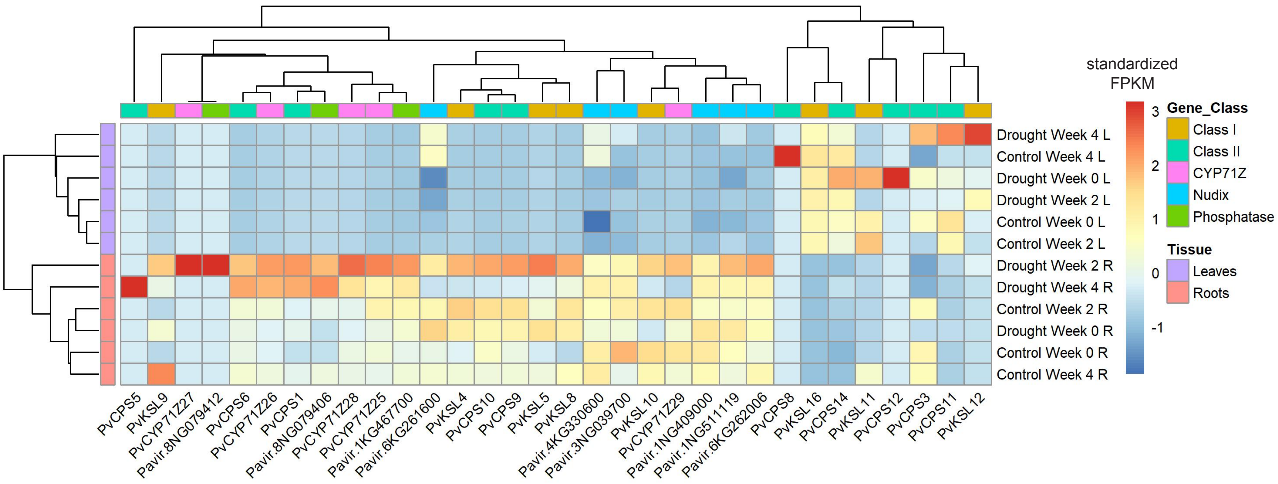

Supplemental Figure 11. Hierarchical cluster analysis of the gene expression of a predicted dolichyldiphosphatase (*Pavir.1KG467700*), two putative phosphoserine phosphatases (*Pavir.8NG079406*, *Pavir.8NG079412*) and putative Nudix hydrolase genes (*Pavir.6KG261600*, *Pavir.4KG330600*, *Pavir.3NG039700*, *Pavir.1NG409000*, *Pavir.1NG511119, Pavir.6KG262006*) as compared to select diTPS and CYP71Z25-29 gene expression profiles from drought stressed switchgrass tissues. Samples were collected before starting treatment (W0), two weeks (W2), and four weeks (W4) of drought stress treatment. L=leaves, R=roots, Drought stressed=D, well-watered=C.
