# Supplementary material for: Cytochrome P450‐catalyzed biosynthesis of furanoditerpenoids in the bioenergy crop switchgrass (Panicum virgatum L.)
Source: Plant J. 2021 Sep 24;108(4):1053–68. doi: 10.1111/tpj.15492 (PMC9292899; doi:10.1111/tpj.15492)
Supplement: Supplementary file 2 — Table S1. Average and standard deviation for each interaction energy (IE) value of filtered docking poses for each P450–substrate combination. Table S2. Synthetic genes used in this study. Table S3. Oligonucleotides used in this study. Table S4. Abbreviations and accession numbers for proteins used for phylogenetic studies. [file TPJ-108-1053-s002.pdf]

**Supplementary Table 1:** Average and standard deviation for each interaction energy (IE) value of filtered docking poses for each P450-substrate combination. The global average IE for docking with the C15-hydroxy and C15,16-dihydroxy substrates suggests that only these two substrate species interact with the P450 active site. CPP = copalyl pyrophosphate.

| Substrate | P450 | Functional Group | | |
|---|---|---|---|---|
| | | C15-pyrophosphate | C15-hydroxy | C15,16-dihydroxy |
| 8,13-CPP | CYP71Z25 | -2.4±0.5 | -6.5±0.5 | -4.8±0.7 |
| | CYP71Z26 | -0.9±0.8 | -4.6±0.8 | -4.5±1.1 |
| | CYP71Z27 | -1.6±0.8 | -5.8±0.8 | -5.2±0.7 |
| | CYP71Z28 | -0.4±0.8 | -6.1±0.4 | -4.3±0.5 |
| | CYP71Z29 | -2.2±0.7 | -5.5±0.4 | -5.0±0.5 |
| (+)-CPP | CYP71Z25 | -1.7±0.6 | -6.3±0.3 | -5.6±0.5 |
| | CYP71Z26 | -0.3±1.2 | -5.8±0.9 | -3.8±0.8 |
| | CYP71Z27 | -1.8±0.5 | -5.9±0.4 | -5.8±0.5 |
| | CYP71Z28 | -0.2±0.6 | -6.0±0.2 | -5.5±0.4 |
| | CYP71Z29 | -1.5±0.8 | -4.7±0.8 | -3.9±0.8 |
| *cis-trans*-CLPP | CYP71Z25 | -1.0±1.1 | -5.6±0.4 | -4.7±0.6 |
| | CYP71Z26 | -0.0±0.9 | -5.3±0.8 | -4.8±0.8 |
| | CYP71Z27 | -1.4±0.5 | -5.2±0.5 | -5.9±0.9 |
| | CYP71Z28 | -0.5±1.0 | -4.8±0.3 | -5.6±0.2 |
| | CYP71Z29 | -1.3±0.9 | -5.6±0.2 | -5.4±0.4 |
| *ent*-CPP | CYP71Z25 | -1.5±0.4 | -6.4±0.2 | -5.6±0.2 |
| | CYP71Z26 | -0.2±0.9 | -5.4±0.7 | -3.6±0.6 |
| | CYP71Z27 | -1.2±0.6 | -6.1±0.5 | -6.2±0.4 |
| | CYP71Z28 | -0.2±0.5 | -5.5±0.4 | -4.2±0.5 |
| | CYP71Z29 | -0.9±1.3 | -5.7±0.7 | -3.6±0.8 |
| *syn*-CPP | CYP71Z25 | -1.7±0.5 | -6.4±0.4 | -5.9±0.6 |
| | CYP71Z26 | -0.1±1.1 | -5.4±0.7 | -5.1±0.7 |
| | CYP71Z27 | -0.9±0.5 | -6.4±0.7 | -6.2±0.5 |
| | CYP71Z28 | -0.4±0.8 | -5.6±0.4 | -5.5±0.4 |
| | CYP71Z29 | -1.0±0.7 | -5.5±0.6 | -5.3±0.4 |
| Average | | -0.9±0.9 | -5.7±0.5 | -5.0±0.8 |

**Supplementary Table 2:** Synthetic genes used in this study.

**>CYP71Z25**
ATGGCTAAAAAGACATCCTCGAAAGGGAAACTCCCCCCAGGGCCTCGCATGCTGCCC
GTAATTGGGAGCTTACACCATCTCTTGAGTTCACCTCTGCTGTTCCGTGTCTTACGGG
GTTTAGCGAAAAAGCATGGGCCACTGATGACATTGCGCTTAGGGGAAGTGCCGGCAC
TGGTTGCGTCAAGCATGGAAGCAGCTCAGGCGATTCTTAAAGTGCACGACACTAATT
TTGCAGATCGGTTCACTCCTGCGACTTTTGCCACCGTCGCTTATGGTGGCACCAATCT
GATATTGTCTCCGTACGGTGAACGGTGGCGGCATTTGAGAAAAATCGTTGTTCAGGA
GATGCTTACTGCAACGCGCGTCCAGAGCTTCAAACATATTAGACAGGAGGAAGTCGG
TCGTTTCTTACACGGAATGGCTGCGAGCGCTGCCGCAGGGACTGCGGTAAACTTCTC
CACAGCAGTATCTAAACTGATTAACGACGCCTTTCTGCGGGAGTGTGTCGGTTCTCGA
TGTAAATATCAGGATGAATTCTTGGACGCCGTTCACACGGCGTCCCTCTTAGCATCTG
GGGTGACAATCGCTGATTTGTATCCCTCTAGTAGAATTATGCAGATGCTGGGAACCGC
ACCTCGTAAAGCCCTGGCGGTAAGACGTCGTGTTGATAGAATCGTTAAACAAATTATC
CAAGAGGCAAAAGAAGCAATGGAGTGTGACGATAAGACTGTCGATGATTCTTTTATT
AGTCTGCTTCTCCGGCTGCAAAAAGAAGGGAGTATGCCGATACCGCTGACGAATGAG
ACAATAATTGCGTTAATGTTCGATATGTTGGCTGCGGGATCCGAAACATCATCAACCAC
CTTGAATTGGGCACTAACTGAACTGATTCGTAGCCCAGCTGCTATGGCGCGCGCCCAA
GCAGAAGTACGTGAAGCTTTCAAAGGAAAAAGTATTATAACGGATGACGACATCGCC
AAATCAGGCATTTCTTACCTAAAACTCGTGTTCAAGGAAACACTGCGTCTGCATCCGT
CCTCACCGTTACTGATTCCGCGCCAATGCCGAGAAACCTGTCAGGTTATGGGCTATGA
TATCCCGAAGGGGACAGCCGTTTTTGTTAACGTGTGGGCAATTGGGAGAGATCCGTT
GTACTGGGAAGATCCTGAAGAATTTAAGCCGGAACGGTTTGAAACGAACAACCTGG
ATTTCAGAGGAACAAACTTTGAGTTCATACCGTTCGGTGCCGGTCGTCGGATGTGTCC
AGGGATTAATCTGGGTCTGGCTAACATCGAACTTGCTTTAGCTTCCCTTTTATATCATTT
TGACTGGAAATTACCGAAAGGTATGGAACCTAAAGATGTTGAAATGCGTGAGGCTGT
AGGTATGGTAGCGAGCAAAGAAACCAGCCTGATTGTCCATCCAGTCACATTTATTCCG
CCTGCCGTTGCGGCGTGA

**>CYP71Z26**
ATGGCGAAAAAAACCTCTAGTAAAGGTAAGTTGCCTCCGGGACCACGGATGCTGCCG
GTTATCGGATCCTTACACCACTTGCTCAGCTCACCGCTGCTGTTCCGAGTTCTGCGGG
GCCTCGCGAAAAAGCATGGGCCTTTGATGACCCTACGATTAGGCGAAGTACCAGCTC
TGGTGGCTAGTTCTATGGAAGCGGCTCAGGCAATTCTCAAAGTGCACGACACGAATT
TTGCAGATCGGTTCACTCCCGCAACGTTTGCCACGGTGGCTTACGGCGGCACAAATTT
GATACTAAGTCCATACGGCGAGCGTTGGCGCCATCTGCGTAAGATTGTTGTTCAGGAG
ATGCTCACAGCCACACGTGTTCAGAGCTTTAAACATATCCGCCAGGAGGAGGTAGGC
AGGTTTTTACAGGGGATGGCAGCATCTGCGGCTGCGGGCACTGCCGTAAACTTTTCC
ACCGCAGTTAGCAAACTGATTAACGACGCTTTCTTACGAGAATGCGTAGGCTCACGTT
GTAAGTATCAAGACGAGTTTCTGGACGCCGTTCATACGGCCAGTCTGCTGGCAAGTG
GGGTCACCATCGCCGATCTCTATCCATCTAGTAGGATTATGCAGATGCTGGGAACAGC
GCCGCGTAAAGCCCTAGCAGTTAGGCGTCGGGTTGACCGCATTGTGAAACAGATTATT
CAGGAAGCAAAAGAAGCGATGGAGTGTGACGATAAAACCGTTGACGACTCTTTTATT
TCGCTGTTACTTCGCTTACAAAAAGAAGGATCGATGCCTATCCCTTTAACCAATGAAA
CAATTATCGCCCTGATGTTTGACATGCTGGCCGCGGGTAGTGAAACTTCCAGTACAAC
CCTGAACTGGGCTTTGACCGAGTTAATTCGCAGTCCGGCTGCCATGGCACGCGCCCA
GGCAGAAGTTCGTGAAGCGTTCAAGGGAAAATCCATCATTACTGATGATGATATTGCA
AAATCGGGTATTTCTTACCTGAAATTGGTGTTCAAGGAAACGCTGAGACTACACCCAT
CATCCCCTCTGCTGATTCCTCGCCAGTGTCGTGAAACCTGTCAGGTGATGGGTTACGA
TATTCCGAAGGGTACTGCTGTATTTGTAAACGTCTGGGCAATTGGTCGCGATCCGTTG

TATTGGGAAGATCCAGAAGAGTTTAAACCTGAACGCTTTGAAACTAATAACCTCGATT
TCCGTGGTACTAACTTCGAATTCATACCTTTTGGTGCGGGAGACGCATGTGTCCGGG
CATAAACCTGGGGCTGGCGAATATTGAGCTAGCGCTGGCGTCTCTACTCTACCATTTC
GATTGGAAGTTGCCGAAAGGTATGGAGCCGAAAGATGTGGAAATGAGAGAAGCCAC
AGGAATGGTAGCTAGCAAAGAAACGAGCCTGATCGTGCATCCCGTAACCTTTATCCCA
CCGGCGGTGGCGTGA

**>CYP71Z27**
ATGGCGAAAAAAACCAGTAGTAAAGGCAAACTTCCTCCGGGTCCACGTATGCTGCCA
GTCATTGGAAGTCTTCATCATTTAGTGTCGTCACCGTTATTGTTTCGTGTACTGAGGGA
ACTGGCTAAAAAACATGGTCCTTTAATGATGTTACGCTTAGGGGAGGTTCCAGCTCTG
GTTGCGAGCTCGATGGAGGCCGCCCAGGCTATTCTTAAGGTTCACGACACGAGCTTC
GCAGATAGATATACACCTGCGACAAGTGCCACCATCGCTTATGGTGGCACTGATCTCA
TTCTGAGCCCTTATGGTGAAAGATGGCGTCACTTGCGCAAAATTGTGGTACAAGAAAT
GCTGACTGCAACGAGAGTTCAGTCCTTTAAGTATATCCGCGAAGAGGAGGTCGCTAG
ATTTTTGCAAGCGATGGCGGCGGCCGCTGCGACAGGGACAGCTGTCGATTTTTCCAC
AGCAGTGGCTAAACTTGTGAACGACGCCTTTCTACGTGAATGTGTAGGTTCCCGGTG
CAAATATCAGGACGAATACTTGGATGCAGTACATACCGTCACCCAGCTGGCCAGCGG
GATAACCATCGCCGATCTGTATCCTTCTTCTCGTATTATGCAAATGCTGGGTACAGCCC
CACGGAAAGGTCTTGCATATCGTCAGCGTATTGACAGAATTTTGAAACAGATCATCCA
GGAAGCGAAAGAAGCTATGGAATGCGAAGACGAGACCGCTCATGAATCTTTTGTAAG
TTTATTACTTCGGCTGCAAAAAGACGGGTCCATGCCTATCCCGCTGACGAACGAAACC
ATAATCGCACTGATGTTTGATATGCTGGCCGCAGGAAGCGACACCTCAAGTACCATTC
TGAACTGGGCGATGACCGAACTGATACGTTCTCCGGCTGCAATGGCACGGGCGCAGG
CAGAGGTTCGAGAAGCTTTTAAAGGTAAATCGATCATTACAGATGACGATATTGCCGA
AAGCGGCATTAGTTATCTTAAACTGGTCTTCAAAGAAACACTGCGTCTGCATCCCACG
TCTCCTTTACTCATACCACGTCAATGTCGGGAGACATGTCAAGTTATGGGGTACGACA
TACCAAAAGGAATGGCAGTTTTTGTCAACGTTTGGGCAATTGGCCGTGATCCAAGCT
ATTGGGATGACCCGGAAGAATTCAAACCGGAACGTTTCGAGACCAATAATCTGGACT
TTCGTGGTACAAACTTTGAATTCATTCCGTTTGGAGCCGGTCGTCGCATGTGTCCAGG
TATAAATCTTGGTCTGGCAAACATCGAACTGGCATTAGCGTCTCTGCTGTATCATTTTG
ATTGGAAACTGCCCAAAGGAATGGAACCGAAGGATGTGGATATGAGAGAAGCAGTA
GGTATTGTTGCATCAAAAGAGACCTCGTTAATTGTACATCCGGTTACGTTTATTCCATT
GACCGTTGCAGCATAA

**>CYP71Z28**
ATGGCCAAAAAGACCAGCAGCAAAGGTAAACTGCCTCCTGGTCCGTGGATGCTGCCG
GTTATTGGTAGCCTGCATCATCTGGTTAGCAGTCCGCTGCTGTTTCGTGTTCTGCGTGG
TCTGGCAAAAAAACATGGTCCGCTGATGACCCTGCGTTTTGGTGAAGTTCCGGCACT
GGTTGCAAGCAGCATGGAAGCAGCACAGGCAATTCTGAAAGTTAATGATACCCGTTT
TGCCGATCGTTTTGCACCGGCAACCAGCGCAACCATTACCTATGGTGCAACCAATCTG
ATTCTGAGCCCGTATGGTGAACGTTGGCGTCAGCTGCGTAAAATTGTTGTTCAAGAAA
TGCTGACCACCACACGTGTTCAGAGCTTTAAACATATTCGTCAAGAAGAAGTTGCCC
GTTTTCTGCAAGGTATGGCAGCAGCAGCCGCAAATGGCACCGCAGTTGATTTTTCAA
CCGCAGTTAGCAAACTGGTGAATGATACCTTTCTGCGTGAATGTGTTGGTAGCCGTTG
TAAATATCAGGATGAATTTCTGGATGCAGTTCATACCGCAAGTCAGCTGGCAAGCGGT
ATTACCATTGCAGATCTGTATCCGAGCAGCCGTATTATGCAGATGCTGGGCACCGCAC
CGCGTAAAGGTCTGGCATGTCGTCAGCGTATTGATCGTATTCTGCGTCAGATTATTCAA
GAGGCAAAAGAAGCAATGGAATGCGACGATAAAACCGCACATGAAAGCTTTGTTAG
CCTGCTGTTACGTCTGCAGAAAGATGGTAGCATGCCGATTCCGCTGACCAATGAAACC
ATTATTGCACTGATGTTTGATATGCTGGCAGCAGGTAGCGATACCAGCAGTACCATTCT
GAATTGGGCAATGACCGAACTGATTCGTAGCCCGACCGCAATGGCACGTGCACAGGC

AGAAGTTCGTCAGGCATTTAAAGGTAAAAGCATCATTACCGATGATGACATGGCCAAA
TCAGGTATCAGCTATCTGAAACTGGTGTTCAAAGAAACCCTGCGTCTGCATCCGACCT
CACCGCTGCTGATTCCGCGTCAGTGTCGTGAAACCTGTCAGGTTATGGGTTATGATATT
CCGAAAGGTACAGCCGTTTTTGTTAATGTTTGGGCAATTGGTCGTGATCCGAGCTATT
GGGAAGATGCAGAAGATTTTAAACCGGAACGCTTTGAAACCAACAATCTGGATTTTC
GTGGCACCAACTTTGAATTTATTCCGTTTGGTGCAGGTCGTCGTATGTGTCCGGGTATT
AATCTGGGTTTAGCAAATATTGAACTGGCACTGGCAAGTCTGCTGTATCATTTTGATTG
GAAACTGCCGAAAGGCATGGAACCGAAAGATGTGGATATGCGCGAAGTTGTTGGTAT
GGTTGCATCAAAAGAAACCAGCCTGACCGTTCATCCGGTTACCTTTATTCCGCCTGCA
GTTGTTTAA

**>CYP71Z25:MRS**
ATGGCTAAAAAGACATCCTCGAAAGGGAAACTCCCCCCAGGGCCTCGCATGCTGCCC
GTAATTGGGAGCTTACACCATCTCTTGAGTTCACCTCTGCTGTTCCGTGTCTTACGGG
GTTTAGCGAAAAAGCATGGGCCACTGATGACATTGCGCTTAGGGGAAGTGCCGGCAC
TGGTTGCGTCAAGCATGGAAGCAGCTCAGGCGATTCTTAAAGTGCACGACACTAATT
TTGCAGATCGGTACACTCCTGCGACTTCTGCCACCATCGCTTATGGTGGCACCGATCT
GATATTGTCTCCGTACGGTGAACGGTGGCGGCATTTGAGAAAAATCGTTGTTCAGGA
GATGCTTACTGCAACGCGCGTCCAGAGCTTCAAACATATTAGACAGGAGGAAGTCGG
TCGTTTCTTACACGGAATGGCTGCGAGCGCTGCCGCAGGGACTGCGGTAAACTTCTC
CACAGCAGTATCTAAACTGATTAACGACGCCTTTCTGCGGGAGTGTGTCGGTTCTCGA
TGTAAATATCAGGATGAATTCTTGGACGCCGTTCACACGGCGACCCAGTTAGCATCTG
GGGTGACAATCGCTGATTTGTATCCCTCTAGTAGAATTATGCAGATGCTGGGAACCGC
ACCTCGTAAAGGCCTGGCGTATAGACAGCGTGTTGATAGAATCCTGAAACAAATTATC
CAAGAGGCAAAAGAAGCAATGGAGTGTGACGATAAGACTGTCGATGATTCTTTTATT
AGTCTGCTTCTCCGGCTGCAAAAAGAAGGGAGTATGCCGATACCGCTGACGAATGAG
ACAATAATTGCGTTAATGTTCGATATGTTGGCTGCGGGATCCGATACATCATCAACCAT
CTTGAATTGGGCAATGACTGAACTGATTCGTAGCCCAGCTGCTATGGCGCGCGCCCAA
GCAGAAGTACGTGAAGCTTTCAAAGGAAAAAGTATTATAACGGATGACGACATCGCC
AAATCAGGCATTTCTTACCTAAAACTCGTGTTCAAGGAAACACTGCGTCTGCATCCGA
CCTCACCGTTACTGATTCCGCGCCAATGCCGAGAAACCTGTCAGGTTATGGGCTATGA
TATCCCGAAGGGGACAGCCGTTTTTGTTAACGTGTGGGCAATTGGGAGAGATCCGTT
GTACTGGGAAGATCCTGAAGAATTTAAGCCGGAACGGTTTGAAACGAACAACCTGG
ATTTCAGAGGAACAAACTTTGAGTTCATACCGTTCGGTGCCGGTCGTCGGATGTGTCC
AGGGATTAATCTGGGTCTGGCTAACATCGAACTTGCTTTAGCTTCCCTTTTATATCATTT
TGACTGGAAATTACCGAAAGGTATGGAACCTAAAGATGTTGAAATGCGTGAGGCTGT
AGGTATCGTAGCGAGCAAAGAAACCAGCCTGATTGTCCATCCAGTCACATTTATTCCG
CCTGCCGTTGCGGCGTGA

**>CYP71Z27:MRS**
ATGGCGAAAAAAACCAGTAGTAAAGGCAAACTTCCTCCGGGTCCACGTATGCTGCCA
GTCATTGGAAGTCTTCATCATTTAGTGTCGTCACCGTTATTGTTTCGTGTACTGAGGGA
ACTGGCTAAAAAACATGGTCCTTTAATGATGTTACGCTTAGGGGAGGTTCCAGCTCTG
GTTGCGAGCTCGATGGAGGCCGCCCAGGCTATTCTTAAGGTTCACGACACGAGCTTC
GCAGATAGATTTACACCTGCGACATTTGCCACCGTCGCTTATGGTGGCACTAATCTCAT
TCTGAGCCCTTATGGTGAAAGATGGCGTCACTTGCGCAAAATTGTGGTACAAGAAAT
GCTGACTGCAACGAGAGTTCAGTCCTTTAAGTATATCCGCGAAGAGGAGGTCGCTAG
ATTTTTGCAAGCGATGGCGGCGGCCGCTGCGACAGGGACAGCTGTCGATTTTTCCAC
AGCAGTGGCTAAACTTGTGAACGACGCCTTTCTACGTGAATGTGTAGGTTCCCGGTG
CAAATATCAGGACGAATACTTGGATGCAGTACATACCGTCTCCCTGCTGGCCAGCGGG
ATAACCATCGCCGATCTGTATCCTTCTTCTCGTATTATGCAAATGCTGGGTACAGCCCC
ACGGAAAGCTCTTGCAGTTCGTCGTCGTATTGACAGAATTGTGAAACAGATCATCCA

GGAAGCGAAAGAAGCTATGGAATGCGAAGACGAGACCGCTCATGAATCTTTTGTAAG
TTTATTACTTCGGCTGCAAAAAGACGGGTCCATGCCTATCCCGCTGACGAACGAAACC
ATAATCGCACTGATGTTTGATATGCTGGCCGCAGGAAGCGAAACCTCAAGTACCACTC
TGAACTGGGCGCTGACCGAACTGATACGTTCTCCGGCTGCAATGGCACGGGCGCAGG
CAGAGGTTCGAGAAGCTTTTAAAGGTAAATCGATCATTACAGATGACGATATTGCCGA
AAGCGGCATTAGTTATCTTAAACTGGTCTTCAAAGAAACACTGCGTCTGCATCCCTCG
TCTCCTTTACTCATACCACGTCAATGTCGGGAGACATGTCAAGTTATGGGGTACGACA
TACCAAAAGGAATGGCAGTTTTTGTCAACGTTTGGGCAATTGGCCGTGATCCAAGCT
ATTGGGATGACCCGGAAGAATTCAAACCGGAACGTTTCGAGACCAATAATCTGGACT
TTCGTGGTACAAACTTTGAATTCATTCCGTTTGGAGCCGGTCGTCGCATGTGTCCAGG
TATAAATCTTGGTCTGGCAAACATCGAACTGGCATTAGCGTCTCTGCTGTATCATTTTG
ATTGGAAACTGCCCAAAGGAATGGAACCGAAGGATGTGGATATGAGAGAAGCAGTA
GGTATGGTTGCATCAAAAGAGACCTCGTTAATTGTACATCCGGTTACGTTTATTCCATT
GACCGTTGCAGCATAA

**Supplementary Table 3:** Oligonucleotides used in this study.

| Primer name | Sequence (with mutated position underlined) |
|---|---|
| CYP71Z25-F86S-F | CGGTTCACTCCTGCGACTTCTGCCACCGTCGCTTAT |
| CYP71Z25-F86S-R | ATAAGCGACGGTGGCAGAAGTCGCAGGAGTGAACCG |
| CYP71Z27-S86F-F | AGATATACACCTGCGACATTTGCCACCATCGCTTAT |
| CYP71Z27-S86F-R | ATAAGCGATGGTGGCAAATGTCGCAGGTGTATATCT |
| CYP71Z25-N95D-F | GTCGCTTATGGTGGCACCGATCTGATATTGTCTCCG |
| CYP71Z25-N95D-R | CGGAGACAATATCAGATCGGTGCCACCATAAGCGAC |
| CYP71Z27-D95N-F | ATCGCTTATGGTGGCACTAATCTCATTCTGAGCCCT |
| CYP71Z27-D95N-R | AGGGCTCAGAATGAGATTAGTGCCACCATAAGCGAT |
| CYP71Z25-E283D-F | ATGTTGGCTGCGGGATCCGATACATCATCAACCACC |
| CYP71Z25-E283D-R | GGTGGTTGATGATGTATCGGATCCCGCAGCCAACAT |
| CYP71Z27-D283E-F | ATGCTGGCCGCAGGAAGCGAAACCTCAAGTACCATT |
| CYP71Z27-D283E-R | AATGGTACTTGAGGTTTCGCTTCCTGCGGCCAGCAT |
| CYP71Z25-T288I-F | TCCGAAACATCATCAACCATCTTGAATTGGGCACTA |
| CYP71Z25-T288I-R | TAGTGCCCAATTCAAGATGGTTGATGATGTTTCGGA |
| CYP71Z27-I288T-F | AGCGACACCTCAAGTACCACTCTGAACTGGGCGATG |
| CYP71Z27-I288T-R | CATCGCCCAGTTCAGAGTGGTACTTGAGGTGTCGCT |
| CYP71Z25-S346T-F | ACACTGCGTCTGCATCCGACCTCACCGTTACTGATT |
| CYP71Z25-S346T-R | AATCAGTAACGGTGAGGTCGGATGCAGACGCAGTGT |
| CYP71Z27-T346S-F | ACACTGCGTCTGCATCCCTCGTCTCCTTTACTCATA |
| CYP71Z27-T346S-R | TATGAGTAAAGGAGACGAGGGATGCAGACGCAGTGT |
| CYP71Z25-S347T-F | CGTCTGCATCCGTCCACTCCGTTACTGATTCCG |
| CYP71Z25-S347T-R | CGGAATCAGTAACGGAGTGGACGGATGCAGACG |
| CYP71Z27-S347T-F | CGTCTGCATCCCACGACTCCTTTACTCATACCA |
| CYP71Z27-S347T-R | TGGTATGAGTAAAGGAGTCGTGGGATGCAGACG |
| CYP71Z25-M463I-F | CGTGAGGCTGTAGGTATTGTAGCGAGCAAAGAA |
| CYP71Z25-M463I-R | TTCTTTGCTCGCTACAATACCTACAGCCTCACG |
| CYP71Z27-I463M-F | AGAGAAGCAGTAGGTATGGTTGCATCAAAAGAG |
| CYP71Z27-I463M-R | CTCTTTTGATGCAACCATACCTACTGCTTCTCT |

**Supplementary Table 4:** Abbreviations and Accession numbers for proteins used for phylogenetic studies.

| Protein name | Species | Accession |
|---|---|---|
| OsCYP71Z6 | *Oryza sativa* | A3A871 |
| OsCYP71Z7 | *Oryza sativa* | Q6YV88 |
| ZmCYP71Z16 | *Zea mays* | Zm00001d014136 |
| ZmCYP71Z18 | *Zea mays* | Zm00001d014134 |
| OsCYP76M6 | *Oryza sativa* | XP_015625955 |
| OsCYP76M7 | *Oryza sativa* | XP_015623593 |
| OsCYP99A3 | *Oryza sativa* | XP_015634021 |
| OsCYP701A6 | *Oryza sativa* | XP_015643248 |
| OsCYP701A8 | *Oryza sativa* | XP_015640905 |
| ZmCYP701A26 | *Zea mays* | Zm00001d046342 |
| ZmCYP701A43 | *Zea mays* | Zm00001d046344 |
| PvCYP71Z25 | *Panicum virgatum* | Pavir.1KG341400[1] |
| PvCYP71Z26 | *Panicum virgatum* | Pavir.1KG382300[1] |
| PvCYP71Z27 | *Panicum virgatum* | Pavir.1KG382400[1] |
| PvCYP71Z28 | *Panicum virgatum* | Pavir.1NG304500[1] |
| PvCYP71Z29 | *Panicum virgatum* | Pavir.1NG309700[1] |
| PhA02218 | *Panicum hallii* | Pahal.1G276500[2] |
| PhA02220 | *Panicum hallii* | Pahal.1G276700[2] |

[1] Accession number for *Panicum virgatum* genome assembly v4.1 (www.phytozome.org)

[2] Accession number for *Panicum hallii* genome assembly v3.1 (www.phytozome.org)
